# Tailored nutrition strategies for Paralympic athletes: addressing unique energy, nutrients, and hydration needs to enhance performance and health

**DOI:** 10.3389/fnut.2025.1572961

**Published:** 2025-07-07

**Authors:** Hadeel Ghazzawi, Raghad Al Aqaili, Batool Khataybeh, Fajer Al-Aittan, Saja Hamaideh, Tamara Alhalaiqah, Adam Amawi, Khaled Trabelsi, Haitham Jahrami, Mahmoud AlHaj Hasan

**Affiliations:** ^1^Department of Nutrition and Food Technology, School of Agriculture, The University of Jordan, Amman, Jordan; ^2^Independent Researcher, Mississauga, Ontario, ON, Canada; ^3^Department of Nutrition and Food Technology, Faculty of Agriculture, Jordan University of Science and Technology, Irbid, Jordan; ^4^Department of Clinical Nutrition and Dietetics, Faculty of Applied Medical Sciences, The Hashemite University, Zarqa, Jordan; ^5^Jordan Paralympic Committee, Amman, Jordan; ^6^Department of Movement Sciences and Sports Training, School of Sport Sciences, The University of Jordan, Amman, Jordan; ^7^Research laboratory Education, Motricité, Sport et Santé, EM2S, LR19JS01, High Institute of Sport and Physical Education of Sfax, University of Sfax, Sfax, Tunisia; ^8^Government Hospitals, Manama, Bahrain; ^9^Department of Psychiatry, College of Medicine and Health Sciences, Arabian Gulf University, Manama, Bahrain; ^10^Hayel Saeed Anam Group, Jeddah, Saudi Arabia

**Keywords:** Paralympic athletes, sports nutrition, nutritional challenges, adaptive sports, disability-specific nutrition

## Abstract

The achievements of Olympic athletes are often highlighted, whereas the successes of Paralympians are frequently overlooked. Paralympic athletes with disabilities face unique nutritional challenges due to variations in energy expenditure and nutrient requirements associated with their specific disabilities and sports. Hydration is critical, particularly for athletes with spinal cord injuries, who may struggle with body temperature regulation. Despite the importance of nutrition in enhancing athletic performance, there is a lack of research focused on the nutritional requirements of Paralympic athletes. This scholarly review provides a comprehensive overview of the energy, macronutrient, and micronutrient requirements, including those for minerals and vitamins, supplements, and fluid intake, of Paralympic athletes aged 18 and older. This literature search was conducted via the Scopus and Google Scholar databases and focused on English-language original articles published between 1990 and 2024. This review included 56 studies. These findings highlight the necessity for tailored nutritional planning to support the performance and health of Paralympic athletes. Close monitoring of individual intake is essential to adjust for fluctuations in macronutrients, micronutrient supplements, and fluid intake. Ongoing research is vital for developing effective nutritional strategies that accommodate the diverse needs of these athletes.

## 1 Introduction

Nutrition is an important factor in improving physical performance and holds significant importance in the lives of competitive athletes (1–3). The terms “Paraplegic” and “Olympic” were first combined to produce the word “Paralympic,” but when the Paralympic Movement became more closely associated with the Olympic Movement, it was modified to include the Greek prepositions “Para” (parallel) and “Olympic” (4, 5). Paralympic (Para) athletes are athletes who are diagnosed with physical, intellectual, or visual disabilities and are categorized according to the International Paralympic Committee (IPC) Classification Code (6). Given the physical challenges and specific needs of this group of athletes, proper nutrition plays a crucial role in supporting their performance and health. The interest in sports for people with disabilities began in 1948 when Dr. Guttmann held the first competition (7). The increasing prominence of Paralympic sports is evident in the recent Paris 2024 Paralympic Games, where as many as 4,400 athletes from around the world participated and competed in 22 sports (8). This significant expansion of Paralympic sports in recent decades underscores the increasing need to understand and address the specific nutritional requirements of para-athletes to optimize their health and athletic potential (9).

There is currently a dearth of studies on the nutrition of para-athletes, although the number of Paralympic sports has significantly increased in recent decades (10). Consequently, swift and collaborative investigations to inform evidence-based nutritional strategies tailored to the evolving landscape of Paralympic sports are urgently needed (11). Para-athletes require tailored nutritional strategies because their disabilities may lead to altered energy expenditure, specific micronutrient needs, or challenges with food preparation and consumption (12). The physiological aspects of disabilities in para-athletes affect their health, nutritional status, training, and performance (13). Regardless of the specific type of disability, the physiological consequences associated with being a para-athlete can increase their susceptibility to micronutrient deficiencies (9). For instance, para-athletes who use wheelchairs frequently exhibit considerable muscle atrophy in their lower limbs, which lowers their lean body mass and makes it more difficult to precisely assess their energy requirements, potentially increasing their risk of micronutrient deficiencies (14). Additionally, para-athletes with spinal cord injuries frequently have lower levels of circulating testosterone, which can have negative impacts on their overall health and athletic performance (15, 16). Variations in muscular strength can also be influenced by their medication use (17).

Given the difficulties in accurately assessing energy requirements in some para-athletes, conditions like relative energy deficiency in sport (RED-S) are a significant concern. RED-S is a syndrome that disproportionately affects para-athletes and is associated with lower bone mineral density and an increased risk of skeletal fractures (16, 18, 19). Research shows that over half of para-athletes have low bone mineral density (20), heightened nutritional needs due to their intense training (21), elevated oxidative stress and inflammation (22). Previous studies have indicated that, often, their food intake does not meet their needs, which can lead to deficiencies in essential nutrients, particularly protein, carbohydrates, vitamin D, and iron (11–13). Low levels of crucial nutrients such as vitamin D and calcium can impair bone mineral density (23). This is particularly significant for para-athletes, including wheelchair basketball players, where maintaining skeletal health is vital for both athletic performance and overall well-being, especially considering the physical demands and potential for falls associated with wheelchair sports (24, 25).

A crucial step in creating an appropriate nutritional strategy to optimize para-athletes’ performance is the evaluation of their nutritional status. Nevertheless, standardized nutritional assessment methods designed for this population have yet to be established (26). The application and interpretation of anthropometric measurements in para-athletes, especially those with physical disabilities, present difficulties (27), and determining the most suitable method for body composition analysis remains problematic (28). In addition, biochemical assessment and exercise blood changes in para-athletes are difficult because of a severe lack of research on this topic (29). Consequently, despite the evident need for tailored nutritional strategies due to the obstacles posed by disabilities, evidence-based nutritional recommendations for para-athletes are lacking (13, 30), and quantifying appropriate macronutrient intakes is complex (31). Furthermore, a knowledge gap exists concerning specific micronutrient requirements and their crucial roles in the bodies of para-athletes (32). This underscores the growing necessity of enhancing dietary knowledge and awareness among para-athletes and developing nutritional guidelines that account for diverse disabilities and suitable learning methods (24).

The fundamental goal of nutritional assessment for Paralympic athletes is to evaluate individual dietary intake, identify deficiencies, and recommend necessary changes to create the most suitable nutritional plan. This plan should consider the nature of the athlete’s disability and the specific requirements of their sporting discipline (3). Compared with Olympic athletes, Paralympic athletes use less muscular energy during exercise (33). However, to guarantee proper nutrition and water consumption, nutritional evaluation is needed (34).

Paralympic athletes require programs that align with their unique characteristics and traits, and they may require greater patience, attention, and nutritional care to achieve their desired benefits. Hence, the approach to this scholarly review aims to provide a comprehensive overview of energy, macronutrients, and micronutrients, including minerals and vitamins, supplements, and liquid intake requirements of Paralympic athletes aged 18 and older.

## 2 Materials and methods

To perform this comprehensive literature review, a comprehensive literature search was undertaken via the Scopus and Google Scholar databases. Following the Preferred Reporting Items for Scholarly Reviews guidelines, we utilized the following query string in the Scopus advanced search: (para OR “para athletics” OR paralympic OR parasport) AND (disabled OR “spinal cord injury” OR tetraplegia OR “vision impairment” OR blind OR “intellectual impairment” OR “coordination impairments” OR “short stature” OR “limb deficiency” OR “impaired muscle power”) AND (macronutrient OR carbohydrate OR protein OR fat OR micronutrient OR mineral OR vitamin) AND (sport OR boccia OR goalball OR shooting OR “sitting volleyball” OR wheelchair) AND “energy” OR “calorie intake” OR “nutrition.” The data search in Google Scholar was performed via keywords such as Paralympic, disabled, athletes, spinal cord injury, wheelchair, nutrition, supplementation, and energy. In addition, we manually reviewed the reference lists of the identified papers, encompassing studies published between January 1990 and October 2024.

### 2.1 Data selection

**Figure 1:**
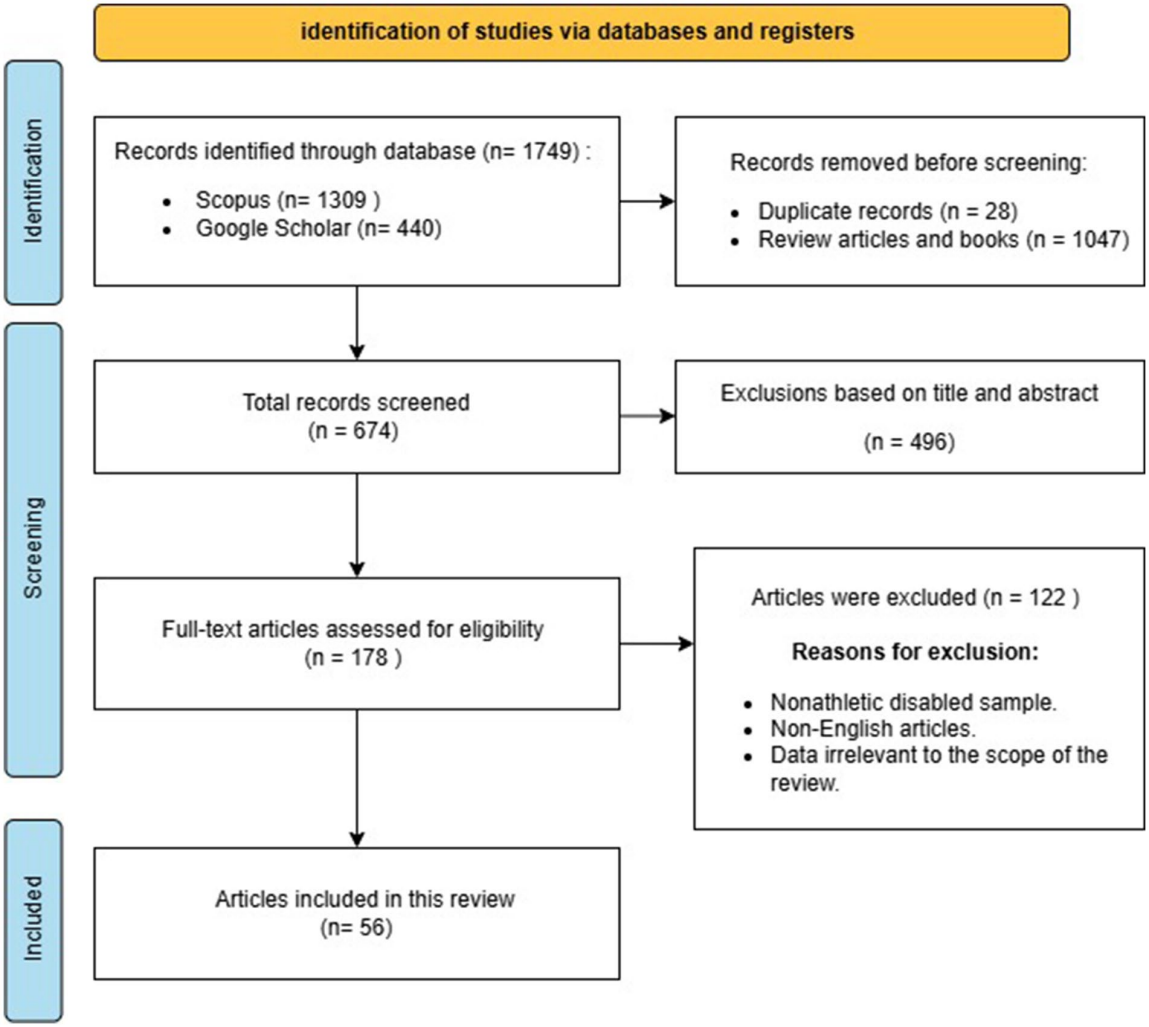
PRISMA diagram with data extraction process and eligibility assessment.

The initial database search identified 1,749 articles related to the nutritional intake, nutritional requirements, and performance and health outcomes of Paralympic athletes. A total of 1,075 articles were excluded because they were duplicate records, reviews, or books. An additional 496 studies were excluded based on title and abstract screening. Subsequently, full-text reviews were conducted for the remaining 178 articles, resulting in 56 studies that met the inclusion criteria. These studies form the basis for the current review. A detailed flowchart of the search strategy is provided in Figure 1.

The inclusion criteria for this review were as follows: (1) original articles that assessed or included disabled male and female athletes over 18 years of age; (2) studies published between 1990 and 2024; (3) articles published in English; and (4) full-text articles.

### 2.2 Data extraction

The titles and abstracts of the identified studies were independently screened by two reviewers according to the inclusion and exclusion criteria, ensuring accuracy and resolving discrepancies to strengthen the reliability of the findings. The full-text articles were then assessed for relevance, with a focus on the role of micronutrients, macronutrients, and supplements and their impact on Paralympic athletes.

### 2.3 Data synthesis

A narrative synthesis was performed to summarize the findings on the nutritional status, knowledge, energy availability, and supplement intake of Paralympic athletes. The results were categorized into energy, macronutrient, micronutrient, and supplementation categories. Each study was examined for key details, including the first author, study design, publication year, sample size, male percentage, and overall conclusion.

## 3 Results

A total of 46 studies met all the inclusion criteria for the final analysis involving 3,633 Paralympic athletes. The reviewed studies explored energy availability, nutritional status, and supplement intake among Paralympic athletes. Nutritional status assessments indicated suboptimal intake of specific micronutrients in athletes, highlighting potential gaps in dietary adequacy. Detailed information on energy availability, nutrient intake, and supplement usage, as well as their outcomes on para-athletes’ performance and health, are summarized in Table 1. The flow diagram of the comprehensive literature search can be found in Figure 1.

**Table 1:** Comprehensive synthesis of articles on energy availability, nutritional status, and supplementation in Paralympic athletes.

No.	Author	Year	Study design	Energy, macronutrient, micronutrient, supplement	Sample size	Mean (age ± SD)	Sex male%	Conclusion
1	Myoenzono (103)	2023	Cross-sectional	Dietary intake and supplement use	1,392	36.1 ± 9.7	75.8%	Paralympic athletes tend to have lower dietary quality and supplement use compared to Olympic athletes, with challenges like meal preparation and consumption and unique energy requirements impacting their diet
2	Hertig-Godeschalk (71)	2023	RCT	CHO, protein, fat	14	34 ± 9	43%	Two female athletes were found to have iron deficiency anemia, and the average vitamin D levels were inadequate.
3	Duarte Junior (56)	2023	Cross-sectional	CHO, protein, fat	30	28.1 ± 8.4	73.3%	Male para-athletes consumed more saturated fat than females, while younger athletes had higher protein intake (g/kg) than older ones
4	Shaw (48)	2024	Cross-sectional	CHO, proteins, fats sodium, cholesterol	31	38	39%	Paracyclists generally meet or exceed nutrient recommendations, but some nutrients fall short, suggesting a need for more dietary variety
5	Islamoglu (52)	2023	Cross-sectional	Energy, nutrient intakes, and EAR	40	N/A	90%	Most basketball players consume inadequate amounts of CHO, fiber, thiamine, Ca, vitamin C, and vitamin D. Limited nutritional knowledge highlights the need for targeted nutrition education for wheelchair basketball players
6	Weijer (83)	2024	Cross-sectional	RMR	67	28 ± 9	55.20%	Fat-free mass, total (T3) levels, and spinal cord disorders are key predictors of RMR in Paralympic athletes. This study’s equations, along with those from Chun et al. and Nightingale & Gorgey, accurately predict RMR in this group
7	Kahveci̇oğlu (31)	2024	Cross-sectional	Energy, macro, micronutrient and hydration intake	66	27.5 ± 9.4	75.8%	Protein, CHO, iron, and Ca intakes fell below recommendations. CHO, vitamin D, Ca, and Mg levels were higher on training days, while hydration was inadequate regardless of gender, sport, or training status
8	Weijer (37)	2024	Cross-sectional	Energy expenditure and nutritional intake	48	27	39.50%	Para-athletes exhibit moderate to high energy expenditure, influenced by exercise duration and fat-free mass
9	Toti (51)	2022	Cross-sectional	Energy, macronutrients, micronutrients, and fiber intakes	15	28.5 ± 1.5	100%	Wheelchair basketball athletes (WBA) often regulate their eating to manage gastrointestinal symptoms and support well-being, including adherence to ON and the Mediterranean diet
10	Deguchi (111)	2021	Cross-sectional	Dietary practice and nutrition knowledge	32	40.5 ± 16.3	68.80%	Para-athletes had significantly lower nutrition knowledge than collegiate athletes. They displayed unique eating perceptions and limited knowledge, despite those practicing healthy eating reporting better body image
11	Sukur (35)	2022	Cross-sectional	Energy intake, protein, carbohydrate, fat, and fluid intake	90	N/A	N/A	Most athletes experience energy intake deficits, with CHO intake often insufficient, while fat and protein intake tend to be excessive. Fluid consumption ranges from 1,000 to 8,000 mL/day
12	AL-Rubaye (81)	2022	Cross-sectional	Energy intake, macronutrient, micronutrient, fiber, cholesterol and caffeine intake	100	34.75 ± 10.13	67%	Despite athletes in this study having anti-inflammatory DII scores, no significant link was found between DII and body composition parameters
13	Madden (36)	2022	Cross-sectional	Macronutrient, micronutrient and supplement intake	80	35.9 ± 8.4	61%	This study emphasizes the need for accurate sources and tailored nutrition education for para-athletes and coaches
14	Gordon (61)	2022	Cross-sectional	Dietary and supplement intake	12	44.0 ± 9.3	83.33%	Intakes of vitamin D and calcium fell below RDA/AI for males. Supplement use was reported at 40% before, 100% during, and 60% after training
15	Yokoyama (112)	2022	A pilot study	Dietary challenges	7	30–70	86%	Wheelchair para-athletes closely monitor calorie intake to maintain mobility, yet many lack routine dietary guidance from dietitians. Some see no need for such advice, often relying on personal notions of an “ideal diet” that may not enhance their performance
16	Hertig-Godeschalk (104)	2022	RCCT	Probiotic and prebiotic supplementation	14	34 ± 9	43%	Implementing a (RCCT) to assess the effect of prebiotic and probiotic supplementation is feasible in elite wheelchair athletes
17	Jeoung (59)	2021	Cross-sectional	Macronutrient and micronutrient intake	21	>18	76%	CHO and protein intake exceeded the %RDA, along with higher intakes of vitamin E, riboflavin, thiamine, B6, and B12
18	Herrera-Amante (26)	2021	Comparative study	Resting metabolic rate (RMR)	15	18.7 ± 6.5	53.33%	The Harris & Benedict equation showed the most consistency compared to indirect calorimetry
19	Baranauskas (62)	2020	Cross-sectional	Energy requirements, macronutrient and micronutrient intake	14	26.4 ± 4.5	0%	Deaf women athletes’ diets are high in fat and saturated fats, increasing their risk of vitamin D and iron deficiencies
20	Sasaki (32)	2021	Cross-sectional	Energy and micronutrient intake	101	33.32 ± 9.88	81.20%	The current Brazilian Federal sports scholarship program fails to adequately prevent micronutrient deficiencies in athletes
21	Egger (49)	2020	Cross-sectional	Energy intake, resting energy expenditure and macronutrient intake	14	34.9 ± 9.4	57.14%	Female athletes have a higher prevalence of low energy availability (LEA) than males, highlighting the need for increased energy intake to support energy demands and training adaptations.
22	Broad (42)	2020	cross-sectional	resting energy expenditure (REE)	14	31 ± 6	100%	The measured REE of wheelchair rugby players was 1735 ± 257 kcal/day. Prediction models for individuals with and without SCI underestimated their REE, likely due to the athletes’ higher REE relative to fat-free mass compared to less active groups
23	Pelly (46)	2018	Case-control	Resting energy expenditure (REE)	7	31.3 ± 7.3	100%	Existing prediction equations for estimating energy needs may need adjustment for athletes with spinal cord injuries
24	Madden (88)	2018	Cross-sectional	Dietary supplement	42	36.3 ± 9.5	78.60%	Supplement use among wheelchair rugby athletes was assessed, with performance and health being key motives. Future studies should examine nutrient intake and physiological levels to determine optimal supplementation techniques
25	Joaquim (40)	2018	Cross-sectional	Energy intake (EI), energy expenditure with exercise (EEex) and energy availability (EA)	17	26 ± 6.17	53%	Paralympic athletes with high exercise energy expenditure (EEex) and insufficient or restricted energy intake (EI) should be closely monitored, as they risk developing low energy availability (LEA) and its effects. Currently, no specific LEA cut-off value exists for this group
26	Joaquim (113)	2019	Cross-sectional	Dietary intake (food groups)	20	N/A	65%	The diet quality of Brazilian Paralympic sprinters during a training camp requires improvements, particularly in wholegrain cereals, dairy, vegetables, and whole fruits.
27	Madden (36)	2017	Cross-sectional	Energy, macronutrient, micronutrient intake, and supplement use	40	20.5–33.5	45%	Females fell short of the RDA for Fe and Ca, while males lacked vitamin A and B9. Common supplements included vitamin D, protein powder, sports bars, and drinks
28	Eskici (114)	2016	Cross-sectional	Energy, macronutrient, micronutrient intake	22	25.5 ± 7.2	0%	This study revealed that some athletes consume insufficient energy, with low CHO intake (42.7%), high fat intake (44%), and inadequate levels of vitamin B1, B9, Mg, Fe, fiber, and fluids
29	Ferro (47)	2017	Cross-sectional	Energy, macronutrient and water intake	11	30 ± 6	100%	Wheelchair basketball players expend less energy than able-bodied athletes but can enhance performance by increasing carbohydrate intake during main meals, especially around training sessions
30	Grams (60)	2016	Longitudinal study	Macronutrient and micronutrient intake	17	30.0 ± 6.5	100%	Elite Spanish wheelchair basketball players’ overall energy intake is positively correlated with their micronutrient intake. A balanced diet with various food groups, especially nutrient-dense options is key to meeting micronutrient needs
31	Flueck (102)	2017	Cross-sectional	Supplement use, fluid and solid food intake	65	39 ± 12	N/A	Swiss wheelchair athletes’ supplement uses closely mirrored that of general Paralympic athletes. The most common supplements included CHO, protein, multivitamins, minerals, recovery drinks, and ergogenic aids like creatine, caffeine, and beetroot juice
32	Krempien (115)	2011	Cross-sectional	Energy, macronutrient and micronutrient intake	32	30.6 ± 6.2	75%	Vitamin and mineral supplements increased men’s nutrient intakes but did not reduce inadequacy rates. These findings highlight those athletes with (SCI) are at risk of multiple nutrient inadequacies compared to the DRIs
33	Goosey-Tolfrey (33)	2010	Cross-sectional	Energy, macronutrient and water intake	23	25.1 ± 8.1 27.6 ± 7.2	39.10%	The energy from CHO, protein, and fat was similar for both F and M groups, though CHO intake was slightly below the recommended level for athletes
34	Potvin (116)	1996	Cross-sectional	Energy, macronutrient and micronutrient intake	10	30.7	100%	Wheelchair athletes nearly met the recommended nutrient intake (RDI) for vitamins and minerals
35	Aiello (53)	2023	Cross-sectional	Energy and macronutrients intake	68	35.7 ± 9.1	100%	Consistent with the link between body composition and energy balance, individuals with (SCI) and skill athletes have lower energy intake per kg of body mass and higher fat mass percentages compared to those in power, mixed metabolism, and endurance sports
36	Gerrish (54)	2017	Cross-sectional	Energy, macronutrient and micronutrient intake	39	21–47	51.30%	There was no significant difference in energy and macronutrient intake between groups based on the severity of the lesion
37	Rastmanesh (117)	2007	Case-control	Nutritional knowledge	72	30 ± 7.6	N/A	Iranian athletes with physical disabilities (APDs) lack essential nutritional knowledge to prevent health issues, particularly regarding disability-specific needs
38	Penggalih (27)	2019	Descriptive	Energy, macronutrients, micronutrients, and fluid intake	18	25	88.90%	The disabled swimmers’ body fat percentage and somatotype differ from professional swimmers’ standards, while their energy, nutrient, and fluid intakes fall short of dietary recommendations
39	Schneider (118)	2023	Cross-sectional	Diet quality	101	N/A	81.20%	Both BHEI-R and GDQS effectively assess diet quality in athletes with disabilities
40	Pritchett (16)	2021	Cross-sectional	Energy availability and macronutrient intake	18	27 ± 7	50%	Calculated energy availability (EA) suggests a low risk of RED-S, but hormonal data indicate a high risk in this para-athlete group
41	Juzwiak (119)	2016	Comparative study	Basal metabolic rate	30	26.5	63.33%	The Owen and Mifflin equations most accurately predicted BMR for these athletes
42	Pegorin (50)	2020	Case study	Energy expenditure with exercise	5	23.6	40%	Athletes with visual impairments showed EEEx levels from light to vigorous intensity
43	Sanz-Quinto (120)	2019	Case report	Nutritional intervention	1	36	100%	The elite wheelchair marathoner training at 3,860 m needs higher nutrient intake and careful adjustments to their nutrition plan
44	Graham-Paulson (97)	2015	Cross-sectional	Nutritional supplements	399	≥18	74%	The main reasons for using nutritional supplements were exercise recovery, immune support, and energy
45	Portela (121)	2024	Cross-sectional	Nutrition knowledge	36	29.8 ± 12.7	72.20%	Athletes’ and coaches’ nutrition knowledge must be improved
46	Gordon (122)	2023	Cross-sectional	Nutrition-related challenges	12	N/A	83.33%	Bowel and bladder problems, restricted hand function, muscle spasms, thermoregulation problems, pressure sores, menstruation, and low iron/anemia were among the physiological and nutritional difficulties that hand cyclists reported. These factors were observed to decrease exercise capacity and restrict food and fluid intake
47	Toti (82)	2021	Cross-sectional	Energy, macronutrients, and micronutrient intake	61	25.4	N/A	Wheelchair basketball athletes improved their diet following dietary advice but did not follow the EAT-Lancet recommendation of high red meat and low legumes
48	Magee (105)	2013	RCT	Vitamin D supplementation	84	≥18	89.30%	This study found a high prevalence of vitamin D deficiency among Irish elite athletes, which also demonstrated that vitamin D3 supplementation in the winter and early spring effectively maintains sufficiency
49	Rodrigues (38)	2018	Longitudinal study	Energy and macronutrients intake	10	29.1 ± 6.06	60%	During the three periods, there were notable differences in body mass, food intake, skinfold thickness, and macronutrients. The most common time for nutritional deficits to emerge is during the pre-competition phase.
50	Amirsasan (80)	2017	cross-sectional	Vitamin and Mineral Intakes	35	37.17 ± 8.69 35.64 ± 12.38	68.60%	Many Paralympic athletes have lower-than-recommended intake of key vitamins and minerals, which are crucial for muscle repair, recovery, and performance. Athletes should focus on improving their diet and food choices
51	Krempien (123)	2012	Cross-sectional	Energy and macronutrient intake	32	30.6 ± 6.2	75%	Athletes with spinal cord injuries face a unique energy balance challenge, balancing energy intake, needs, and expenditure during physical training. They may monitor or restrict their diet to prevent obesity
52	Graham-Paulson (106)	2018	Case study	Caffeine	1	46	100%	Caffeine enhanced the 20 km TT performance of an elite male Para-triathlete, likely due to increased arousal and higher power output at the same RPE
53	Gawlik (124)	2015	Cross-sectional	Lipid profile	32	29 ± 9.03	100%	The study participants had a lower percentage of overweight and obese men compared to the general male population in Poland, along with a better lipid profile
54	Pritchett (125)	2016	Longitudinal study	Vitamin D	39	27.7 ± 6.5	48.70%	In the autumn and winter, a significant percentage of top athletes with SCI had deficient (15.4%) and insufficient (41–51%) 25(OH)D levels. However, this investigation did not find any seasonal drop in vitamin D level
55	Flueck (98)	2014	RCT	Caffeine and sodium citrate	9	28	66.67%	There was no ergogenic effect of caffeine and/or sodium citrate supplementation on the 1,500-meter workout performance of wheelchair top athletes
56	Spendiff (99)	2005	RCT	Glucose drink	8	31 + 5	100%	Both high and low carbohydrate intake prior to exercise did not result in a hypoglycemic reaction or negatively impacted exercise and performance in a group of paraplegic athletes with modest lesion severity

### 3.1 Energy requirements

Multiple factors influence the energy requirements of athletes with disabilities, such as body composition, the training phase, the equipment used during training or competition, and, importantly, the type of impairment (31, 35, 36). These factors result from the differences in physiological and metabolic functions between these individuals and their nondisabled peers, including variations in muscle mass, spasticity, and thermoregulation (36).

#### 3.1.1 Energy demands by sport and classification

Energy expenditure varies greatly across sports and classifications, reflecting different physiological demands. For example, one study reported that athletes with spinal injury had relatively lower total daily energy expenditure (TDEE) values (2,379 ± 522 kcal/day), followed by athletes with limb deficiencies (3,140 ± 837 kcal/day) and athletes who were visually impaired (3,882 ± 1,003 kcal/day). These differences are attributed to differences in muscle activity, metabolic demands, and mobility across impairments, emphasizing the critical role of classification in determining energy needs (37). This finding is in line with the work of Rodrigues et al. (38), who reported some remarkable fluctuations in the energy intake of Paralympic track and field athletes across three phases: end-of-season, post-vacation, and precompetition. At the end of the season, the daily energy intake of athletes ranged between 1,667 and 2,578 kcal, which reflects a decrease in training demand. Postvacation, energy intake varied widely, with seven athletes increasing their intake by as much as 783 kcal/day and three athletes decreasing their intake by up to 703 kcal/day. This phase likely reflects adjustments to reduced physical activity and compensatory dietary habits. During the precompetition phase, energy intake changes were carefully matched to performance demands, with three athletes increasing their intake to 644 kcal/day and five athletes decreasing their intake in an effort to support an optimized body composition for competition, some by as much as 704 kcal/day.

The challenge of maintaining adequate energy availability (EA) in Paralympic athletes has been underscored by a study showing that 82.3% of athletes with visual impairment (VI), cerebral palsy (CP), and limb deficiency (LD) exhibit EA levels below the recommended minimum of ≥45 kcal/kg of fat-free mass (FFM) per day. Energy availability refers to the amount of dietary energy remaining for basic physiological functions after accounting for the energy expended during exercise. It is typically expressed in kilocalories per kilogram of FFM (kcal/kg FFM) (39). Inadequate EA can lead to a range of adverse outcomes, including impaired athletic performance, compromised immune function, menstrual disturbances, and long-term health consequences such as reduced bone mineral density (40).

#### 3.1.2 Resting metabolic rate and total energy expenditure

The total energy expenditure (TEE) refers to the energy produced by the demand of metabolic processes and consists of the sum of three components: resting metabolic rate (RMR), energy expenditure with exercise (EEEx), and the thermic effect of feeding (TEF) (41). Among these, the RMR plays a crucial role in estimating TEE, as it represents the amount of energy required to sustain basic physiological functions at rest (26). In Paralympic athletes, RMR varies significantly due to factors such as reduced muscle mass and, in some cases, alterations in autonomic nervous system function (31). Numerous studies have documented the lower RMR and overall energy expenditure observed in this population, underscoring the need for individualized energy assessments (36). For example, a study reported that the average RMR for female athletes was 1,386 ± 258 kcal/day and that for male athletes was 1,686 ± 302 kcal/day; the RMR was significantly different between the impairment groups. Those athletes with spinal cord disabilities (SCDs) exhibited significantly lower RMRs (1,366 ± 241 kcal/day) than those with either visual or hearing impairments (1,744 ± 347 kcal/day), and this reduction stems from muscle atrophy and impaired activity of the sympathetic nervous system (SNS) in SCI, thereby decreasing catecholamines, hormones that lead to metabolic processes such as thermogenesis (42), and many researchers have suggested that a decrease in SNS activation can result in an RMR decrease of 4 to 9% (43–45). A study of Canadian elite wheelchair rugby and basketball players with spinal cord injury (SCI) reported a daily energy intake of 2,115 ± 420 kcal on average (42). The comparatively low intake was explained by the atrophy of lean muscle mass and metabolic adaptations after SCI, which collectively reduce the total energy expenditure needed for body weight maintenance (42). Moreover, other indirect calorimetry studies have attempted to develop and validate models for accurate RMR prediction. For example, a study measured resting energy expenditure (REE) in SCI male athletes and reported that the average REE of these athletes was 6,437 kJ/day (approximately 1,538 kcal/day). More interestingly, SCI athletes had a significantly greater REE per kilogram of lean tissue mass (LTM), with 146 kJ/kg LTM/day, than the 125 kJ/kg LTM/day in able-bodied controls. Although the study did not report athletes’ total daily energy intake (EI), it emphasized the importance of accurately estimating REE for energy balance, since traditional REE prediction models tend to underestimate the actual caloric requirements for athletes with SCI, necessitating customized energy assessments (46).

Finally, another study raises important considerations toward a valid assessment and prescription of the energy needs of top-level wheelchair basketball players, detailing complications in estimating energy expenditure (EE) and unique physiological factors affecting these athletes. Although the average daily caloric intake was 2,492 ± 362 kcal in May and 2,470 ± 497 kcal in June, the estimated EE calculations produced values of 3,591 ± 711 kcal and 3,791 ± 732 kcal, respectively, indicating a deficit of approximately 1,000 kcal/day. However, athletes maintained stable body weights, suggesting that EE values may be overestimated because of reliance on compendiums designed for able-bodied populations (47).

#### 3.1.3 Gender-based energy consumption in Paralympic athletes

Gender-specific considerations in estimating energy requirements have gained attention in sports nutrition research, even among able-bodied athletes. Kahvecioğlu and Aktaç (31) reported average daily training energy intakes of 1577.6 ± 549.8 kcal for males and 1447.1 ± 492.5 kcal for females, suggesting that female athletes may be at greater risk of low energy availability (LEA). LEA arises when energy intake is insufficient to meet both exercise demands and basic physiological functions, often defined as ≤30 kcal/kg fat-free mass/day. It can lead to hormonal disruptions, menstrual irregularities, reduced bone density, and impaired performance, underscoring the need for individualized, sex-specific nutrition strategies. Interestingly, the same trends have been observed in elite para-athletes. Shaw et al. (48) described male paracyclists (2,878 kcal/day) as having significantly higher energy intakes than female paracyclists (1,760 kcal/day), suggesting the need for sex-specific individualized dietary planning within both Paralympic and able-bodied athlete cohorts. A study reported that female wheelchair athletes reported low energy availability on 73% of the training days, whereas in men, the figure was only 30%, primarily because energy intake is lower in female athletes (1,377 versus 2,276 kcal/day). This focuses on the important need to establish sufficient energy input in female competitors to avoid the unfavorable consequences of LEA on recuperation, immune function, and performance (49). In a study on Brazilian Paralympic sprinters with visual impairments, session-specific energy expenditure due to exercise (EEEx) during a four-day preparatory training period ranged between 190 and 380 kcal. The EEEx of male athletes was between 238 and 380 kcal per session, whereas that of female athletes was between 190 and 340 kcal, depending on the intensity of exercise. The intensity varied from 1.3 to 21.5 metabolic equivalents (METs), reflecting a light to vigorous range of activity corresponding with the stresses of sprinting. The results underline the necessity of more precise adaptations concerning energy intake so that an optimal energy balance is maintained for both genders (50). Another study examined the risk of low energy intake, low energy availability (LEA), and relative energy deficiency in sport (RED-S) in 18 national and international Paralympic athletes and reported that the energy intake was 1,717 ± 280 kcal/day for females and 2,566 ± 651 kcal/day for males. Although the EA could only be calculated in four male athletes due to incomplete data, none of these had a mean EA below the LEA threshold of 25 kcal/kg FFM/day. Female athletes demonstrated large day-to-day EA variability, including some who exhibited LEA on some days, but the mean EA remained above the LEA threshold (16). Notably, sex differences in energy consumption were reported by Madden et al., as female athletes consistently exhibit lower energy intake than their male counterparts do (4).

#### 3.1.4 Challenges in achieving energy balance

Nevertheless, some studies suggest that Paralympic athletes reach their recommendation of energy intake, such as wheelchair basketball athletes (WBA), with a total energy intake of approximately 32.6 kcal/kg of body mass/day—similar to the levels observed in able-bodied active individuals. Elevated energy consumption was allocated primarily to support the athletes’ rigorous training schedules, which averaged nearly 15 h per week (51). To support this assertion, a study investigated the dietary intake of wheelchair basketball athletes (WBA) and reported a daily average caloric intake of 1967.68 kcal. This value is within the broad recommended range of 1,500–2,300 kcal a day for top athletes and indicates that, at a minimum, these athletes definitely fulfill their minimum energy requirements on average (52). Even though they comply with the general guidelines, the majority of Paralympic athletes do not achieve an adequate energy balance. For example, a study reported that female wheelchair basketball players ingested (2,363 ± 905 kcal/day), yet their measured energy expenditure was considerably greater (2,908 ± 797 kcal/day), indicating a high energy deficit (37). Similarly, another study revealed that Paralympic athletes who are on a scholarship have a lower energy intake (2,128 kcal/day) than those without a scholarship (2,239 kcal/day). These disparities can negatively impact the effectiveness of training, compromise recovery processes, and increase the risk of micronutrient deficiency and chronic low energy availability (32).

Notably, discrepancies in energy balance exist, particularly among athletes with SCI and those with other health conditions. Research has shown that Paralympic athletes with a diagnosis of SCI have significantly lower caloric intake (1,978 kcal/day) than athletes with other health conditions (2,263 kcal/day), despite having similar energy requirements, which could be associated with different factors, such as medication side effects, reduced appetite, and impaired nutrient absorption (53).

Additional variability in energy balance has been reported in a study of elite American and Canadian athletes with SCI; although the macronutrient distribution largely fell within the recommended ranges, total caloric intake was consistently low, at means of 1,602 kcal/day for females and 1,893 kcal/day for males during the autumn, and these values decreased even further during winter, to 1,463 kcal/day for females and 1,669 kcal/day for males. These values are below the 1,800 kcal/day normally recommended for high-performance athletes, indicating that SCI athletes may not be meeting the energy requirements for intensive training (54). These figures are less than the 1,800 kcal/day recommended for high-level performing athletes and suggest that wheelchair swimmers or swimmers with disabilities may be neglecting sufficient energy under heavy training conditions. In the precompetition phase, when their training loads were greatest, a study also revealed that the average energy need of swimmers in this phase was 2,969 ± 473 kcal/day, whereas actual intake was met at only 1,627 ± 632 kcal/day, which supported only 55.5% of their requirements. This high deficit can undermine recovery and adjustment to the demands of training (27). Last, the control of energy intake constitutes a complicated matter, particularly in athletes prone to inappropriate weight gain and associated metabolic issues. For example, it has been reported that some wheelchair basketball athletes (WBAs) have a daily energy intake as high as 2867.8 ± 523.6 kcal, which, if not balanced with their real expenditure of energy, can put them at risk of undesired weight gain and worse sport performance (25).

#### 3.1.5 Practical recommendations for energy balance

In general, maintaining energy balance is a significant challenge for Paralympic athletes, specifically those with high energy needs; frequent monitoring and an individualized dietary strategy are recommended by many studies. In some cases, individualized nutrition plans are needed to control Paralympic athlete-specific health problems, such as neurogenic bowel dysfunction (NBD), in wheelchair basketball athletes (WBA). Regular nutrition follow-up in such cases not only allows for individualized nutrient intake but also helps reduce symptoms, normalize gastrointestinal function, and improve the overall quality of life. This approach guarantees that nutritional interventions are both effective and responsive to the evolving medical and performance needs of the athlete (40), and a study underlines the repeated need for customized nutritional plans for Paralympic athletes to maintain their energy needs, with a special focus on pre- and postexercise nutrition (49). In specific cases, individualized dietary strategies can address health conditions unique to Paralympic athletes, such as neurogenic bowel dysfunction (NBD) in WBA, ensuring that nutritional interventions are both effective and targeted (51). Accurate REE assessments are critical in developing precise dietary plans to prevent negative energy balance (46), and understanding REE helps practitioners develop nutritional plans to meet the unique demands of training, ensuring that athletes avoid risks of energy imbalance that may impair recovery, reduce performance, or predispose them to injury (42).

### 3.2 Carbohydrates

The essential main energy source during high-intensity exercise is carbohydrates (27). High-intensity exercise depletes muscle glycogen reserves (55). Poor carbohydrate intake has negative effects, such as a greater risk of muscle cramps and injury, a greater risk of fatigue, and a decrease in athlete performance (27, 38). Perioding carbohydrate intake on the basis of exercise load or personal objectives is necessary for athletes (38). There are no set recommendations for carbohydrate intake in para-athletes (56), as studies involving paracyclists with SCI have shown that competition and exercise cause comparatively high levels of carbohydrate oxidation (13). Since carbohydrates are the primary energy source used during exercise, they must account for a sizeable portion of the diet’s overall energy content (56, 57).

The International Society of Sports Nutrition recommends that athletes who engage in moderately intensive training continually replenish their muscle and liver glycogen reserves by eating 5 to 8 g/kg body weight daily (56). Since active muscle, a key glycogen reservoir for storing, is typically lower in Para athletes who suffer from SCI, amputations, or cerebral palsy (13, 56), Para athletes could have reduced glycogen stores once they begin exercising. As a result, higher carbohydrate consumption than that recommended for healthy-bodied athletes might be beneficial (58). Glycogen regeneration and storage in athletes are significantly influenced by carbohydrates. For example, despite the origin of their condition, wheelchair athletes make extensive use of their upper body muscles; however, they utilize the same amount of muscle glycogen as athletes without disabilities do, despite having far less stored muscle glycogen. As a result, it is advised to consume more carbohydrates as a preworkout (59).

Although wheelchair athletes use glycogen similarly to healthy athletes do, their glycogen levels are typically low when they first start exercising. Therefore, a greater amount of carbohydrates should be consumed prior to exercise to restore glycogen stores and prevent performance loss (33). However, according to one study, wheelchair athletes who consume too many simple carbs may experience movement restrictions as a result of fat buildup around their waists (60). For endurance hand cyclists with SCI, the suggested carbohydrate consumption before and after training is 1–4 g/kg body weight and 1–1.2 g/kg body weight, respectively. Additionally, the recommended carbohydrate consumption during training is 30–60 g per hour of exercise (61). In addition, Indonesian athletes with disabilities consume an average of 279.96 g of carbohydrates per day, which suggests that their consumption of carbohydrates is inadequate given their nutritional requirements (35). Moreover, the carbohydrate intake for deaf women’s basketball players was 5.0 ± 1.3 g/kg of body weight, whereas the recommended intake is 5–8 g/kg of body weight, indicating that those athletes simply consumed the minimal amounts of carbohydrates that were advised (62). Additionally, a study by Egger and Flueck (49) revealed that the daily intake of carbohydrates for male and female Wheelchair athletes was 4.0 g kg^−1^ body mass and 2.4 g kg^−1^ body mass, respectively, while the recommendation was ≥3 g kg^−1^ body mass, indicating that males only met their requirements.

In terms of fiber, the current macronutrient consumption limits for Paralympic competitors are mostly based on the dietary guidelines for athletes with disabilities. Consequently, Paralympic athletes should ingest 3 to 5 g kg^−1^ body mass of carbohydrates per day while training at a low volume and 8 to 12 g kg^−1^ body mass per day when exercising at a high volume (37). To prevent gastrointestinal problems (such constipation) and improve athletic performance, Paralympic competitors are encouraged to maintain a high-fiber diet (62). All people with disabilities, regardless of whether they are athletes or not, frequently consume insufficient amounts of dietary fiber (52, 63). For Paralympic athletes, the intestinal passage may last up to 80 h; hence, a high-fiber diet is advised to control bowel motions (52, 64). Furthermore, because wheelchair athletes experience a high rate of constipation, adequate fiber intake is particularly crucial for intestinal motility in these athletes (65). To control fecal bulk and reduce the risk of hypercholesterolemia and metabolic disorders, adequate fiber consumption is crucial (48). This can also help athletes suffering from SCI overcome constipation (66, 67). However, consuming too much fiber might worsen symptoms; therefore, intake must be customized as needed to attain optimal bowel function (48, 68). For example, it has been proposed that individuals with SCI consume 18–31 g of fiber daily (69), which is less than the present Dietary Reference Intakes (DRIs) for males (38 g/day) but compatible with DRIs for females (25 g/day) (48).

To the best of the authors’ knowledge, there is no recommended daily intake of fiber for Paralympic athletes. However, given the potential harm that disability may cause to the gastrointestinal tract’s capacity to operate, frequent fiber ingestion is highly advised (56, 58). For example, certain gastrointestinal issues may be present in para-athletes who have had SCI (47). As a result, dietary programming is necessary to ensure that fiber intake is neither too high nor too low to preserve the normal function of the gastrointestinal tract (56).

### 3.3 Protein

The consumption of protein is necessary for the production of hormones and enzymes, tissue repair, and protein breakdown compensation since exercise increases protein catabolism (27, 31). If adequate, protein was not consumed. The critical amino acids, particularly leucine, support muscle protein synthesis during the recovery phase; in that case, protein catabolism and muscle and body protein degradation prevail, resulting in muscle mass loss and a negative nitrogen balance (52, 62, 70). Protein needs and consumption after exercise have been estimated to be equivalent to those of athletes without impairments (52). If the athlete has a pressure wound, the demand may increase slightly (52). Therefore, muscle atrophy and a negative nitrogen balance can arise from the impact of dietary proteins on muscle protein synthesis throughout the healing phase (59). Because there are no guidelines for athletes with disabilities, their post exercise protein needs and consumption are comparable to those of athletes with impairments (58, 59). Athletes must consume enough protein each day (>1.2 g/kg body mass) to promote adaptations to training and aid in recuperation during all training periods, particularly to compensate for the elevated level of muscle protein breakdown (71). However, another investigation indicated that athletes with disabilities consume less protein than athletes without disabilities since they have less muscle activity and fewer energy needs (59, 72). The Recommended Dietary Allowance (RDA) for the able-bodied population is 0.8 g/kg body mass daily, according to Thomas et al. (73), but the daily requirement for protein among able-bodied athletes falls between 1.2 and 2.0 g/kg body mass (59). Moreover, a study by Jäger et al. (74) reported that a protein intake of 1.4–2.0 g/kg of body weight is advised for healthy athletes. According to Ferro et al. (47), the recommended daily intake of protein for Paralympic athletes varies and ranges from 1.4 to 1.6 g/kg of body weight. Weijer et al. (37) stated that Paralympic athletes should eat between 1.2 and 1.8 g kg^−1^ body mass per day. According to Lee et al. (75), elite athletes with disability recovery should consume 1–1.5 g/kg protein daily. However, Paralympic athletes might additionally need a high-protein diet to aid in muscle development and repair (56), and research on the protein requirements of para-athletes is scarce. Flueck and Parnell (72) recommended a protein intake of 1.2 g/kg of body weight per day for athletes with spinal cord injury (SCI), extrapolating from data on able-bodied athletes. They also noted that certain conditions—such as renal impairment, constipation, or a significant reduction in active muscle mass—may necessitate lower protein intake in this population. Athletes with SCI who are following diets low in calories to gain lean muscle or who have greater levels of active muscle, an ulcer in the abdomen, or slower stomach emptying are recommended to consume more protein (56). Like carbohydrates (CHO), the daily protein requirements for athletes with SCI and other para-athletes are currently quite comparable to those for athletes without SCI; however, adequate protein intake is required to maximize the production and repair of muscle proteins, particularly following exercise (61).

For endurance hand cyclists with SCI, consuming 0.3–0.5 g of protein per kg of body weight is recommended within 30 min postexercise (61). According to Baranauskas et al. (62), deaf female athletes consume protein at approximately 1.3 ± 0.3 g/kg of body weight. However, as a recommendation for deaf athletes, the suggested protein intake must be 1.4–1.6 g/kg of body weight (62). A study by Egger and Flueck (49) revealed that the daily intake of protein for male and female wheelchair athletes was 1.4 g kg^−1^ body weight and 1.1 g kg^−1^ body weight, respectively, whereas the recommendation was ≥1.2 g kg^−1^ body weight, indicating that males met their requirements with little variation with respect to females (49).

### 3.4 Fat

Dietary fats are crucial for both health and athletic performance and are considered vital components of an athlete’s health (56) because they offer body strength, energy production, organ preservation, and hormone synthesis; help the body absorb fat-soluble vitamins and fatty acids; and are a part of cell membranes (31, 38, 56). According to previous studies, fat should not constitute more than 25 to 30% of the total energy content of para-athletes’ diets (57, 58). It is advised that athletes with disabilities consume between 28 and 37% of their calories from fat (52, 63). Studies carried out by Eskici and Ersoy (25) and Islamoglu et al. (52) revealed that wheelchair basketball athletes consumed 44 and 43% fat from their total energy, respectively, while the recommended fat intake is between 28 and 37% (52); however, on the basis of meal records, it might be assumed that the high fat intake could be caused by the fact that they typically ate red meat, consumed many nuts and oilseeds, and sometimes used fat to compensate for the carbohydrate deficit (52). Moreover, Indonesian Paralympic athletes consume an average of 80.66 g of fat per day, which is excessive compared with their dietary requirements (35).

The improvement in athletes’ general health and fitness levels is closely related to eating the right diet. The scientific data indicate that athletes with disabilities—in addition to those who are deaf—consume too much fat (62). For deaf female athletes, in terms of caloric requirements, the ideal range of fat is 20–35%, which can be explained as follows: 6–10% polyunsaturated fatty acids, 1–2% omega-3 fatty acids, 5–8% omega-6 fatty acids, and less than 10% saturated fatty acids (62). However, Baranauskas et al. (62) reported that females consumed approximately 37% fat, which is above the maximum amount of the recommended intake. In addition, athletes with disabilities, such as those in wheelchairs or those who have suffered spinal cord injuries, consume between 29 and 44% of their calories from fat (25). Epidemiological evidence shows that a high-fat diet increases the likelihood of obesity and cardiovascular mortality in a certain population that is not physically active (76). However, there is no scientific evidence that foods rich in fat cause athletes to gain weight or increase cholesterol. Nonetheless, there is a link between high-fat diets and the body’s homocysteine levels, which encourages the development of cardiovascular disorders (62).

Interestingly, polyunsaturated and monounsaturated fats have anti-inflammatory properties that reduce inflammation in a variety of physiological and pathologic circumstances (56, 77). Furthermore, polyunsaturated fats, such as v-3 fatty acids, can improve skeletal muscle anabolism by preventing wasting and accelerating healing (56).

### 3.5 Vitamins

#### 3.5.1 Vitamin D

Vitamin D deficiency is a concern of major importance in Paralympic athletes, particularly those with SCI (71) and those competing in wheelchair sports (51). The consequences of poor vitamin D are widespread, affecting not only bone health but also immune function and overall athletic performance (51). Many studies have investigated the prevalence of insufficient vitamin D and its causes in these populations, highlighting that it is a persistent concern in many different disciplines (71).

One of the findings was the lack of Sports Nutrition knowledge (SNL) on vitamin D among 49 SCI athletes and 24 coaches, and the study revealed that only 42% of participants were able to identify fatty fish as a good vitamin D nutritional source, despite the crucial role of this vitamin in calcium regulation and bone health (78). This knowledge gap is of concern, as athletes with SCI are already at greater risk for vitamin D insufficiency or deficiency associated with a reduction in sun exposure and altered metabolic activity (78).

Many studies suggest suboptimal dietary vitamin D levels in athletes with SCI. For example, a study examining the total nutritional intake of 39 elite SCI athletes from the United States and Canada reported that these athletes consumed an average of 0.8 μg/day of vitamin D, which is only 5% of the recommended adequate daily intake of 15 μg. This was largely attributed to insufficient energy intake, which was insufficient to meet the micronutrient requirements of these individuals. Further analyses revealed that this low level of intake occurred in the winter months due to low sun exposure (27). These findings are similar to those of a study of South African SCI endurance athletes, which reported that males had a median intake of 2.4 μg/day (69% of the RDA), whereas female athletes had an intake of 5.1 μg/day (102.5% of the RDA). Even though females meet the RDA, these levels are still thought to be insufficient, especially for SCI athletes, who are already at risk for low Bone mineral density (BMD) because of their lack of weight-bearing activity (61). Bone mineral density (BMD) is a measure of the amount of mineral matter per square centimeter of bones, serving as a key indicator of bone strength and fracture risk. Low BMD, encompassing conditions such as osteopenia and osteoporosis, reflects diminished bone mass and structural deterioration, making bones more susceptible to fractures. In individuals with SCI, mechanical unloading from paralysis leads to rapid and significant loss of BMD, particularly in the lower limbs, increasing the likelihood of fragility fractures (79).

Further evidence of vitamin D insufficiency comes from a study on Lithuanian high-performance deaf female basketball players, where blood tests revealed that only 14.3% of athletes presented sufficient levels of vitamin D, with a mean serum 25(OH)D concentration of 24.1 nmol/L—below the threshold ensuring adequate bone health (62). Moreover, in a study with Brazilian para-athletes, vitamin D intake was consistently below the RDA; males consumed an average of 9.3 μg/day, whereas females consumed 11.1 μg/day on average (32). While the female athletes’ intake was somewhat higher, it was still not enough to provide optimal bone health and immune system functioning, especially for athletes who trained during times when sun exposure was scarce (62).

A study that evaluated the dietary intake of 35 elite Paralympic athletes in Iran demonstrated that each gender has a low intake of vitamin D; males only averaged 1.45 ± 1.30 μg/day, representing 9.78% of the recommended dietary allowance, whereas females only averaged 1.16 ± 0.80 μg/day, representing 7.37% of the RDA. These deficiencies were due to the athletes’ dependence on a limited number of sources, which turned out to be inadequate to meet their needs (80). A more inclusive study, however, involving a sample size of 100 Paralympic athletes—48 undergoing hemodialysis and 52 with hemophilia—exhibited a very low mean intake of vitamin D at 2.55 ± 5.007 μg/day, which is below the recommended range of 10–15 μg/day. This is especially concerning for athletes on hemodialysis, as they are at greater risk for developing osteodystrophy due to impaired calcium metabolism (81).

Moreover, a large-scale study of 66 Paralympic athletes from swimming and wheelchair basketball revealed more evidence for vitamin D insufficiency. The results revealed that female athletes had a mean intake of 5.1 μg/day on training days, whereas male athletes consumed only 2.7 μg/day, which further decreased on nontraining days. Inadequate levels of vitamin D intake are alarming, particularly because vitamin D plays a vital role in calcium absorption, maintaining bone health, and supporting immune function, especially among athletes who participate in demanding physical activities (31). The chronic inadequacy of vitamin D intake among Paralympic athletes could result in long-term health complications such as diminished bone mineral density, immune malfunction, and poor performance (23).

#### 3.5.2 Vitamin A

Vitamin A plays crucial roles in immune function, vision, and general health (61). Various studies have assessed vitamin A intake in disabled athletes, and the evidence is quite mixed, given the dependency on the at-risk group and an individual’s needs (51).

Among the population of wheelchair basketball athletes (WBAs), the mean vitamin A intake (928 ± 184 μg/day) exceeded the RDA. This finding suggests that, overall, these athletes are meeting their dietary needs, although there are some significant individual variations in the adequacy of nutrient intake (51). Further study revealed that male athletes consumed a median of 1810.5 μg/day (201.7% of the RDA), whereas females reported a higher intake of 2886.9 μg/day (412.4% of the RDA), reflecting a diet rich in vitamin A sources such as vegetables and fortified dairy products (61). However, a study on Indonesian swimming athletes revealed that the mean intake of vitamin A was 232 μg/day, which is only 39% of the daily recommended intake of 600 μg. This relatively low intake of vitamin A—rich foods, such as vegetables and fruits (27). Similarly, Paralympic athletes in Tabriz, Iran, presented notable deficiencies in vitamin A, with females consuming only 508.87 ± 217.40 μg/day (72.12% of the RDA) (80). Conversely, Brazilian para-athletes, especially those in high-performance categories, showed exceptional intake of vitamin A, with a median consumption of 11,330 IU/day, far above the RDA of 3,000 IU/day. This high intake was explained by their dietary profiles, which included fortified foods that were rich in vitamin A, as well as vegetables and animal-derived products (56) Athletes involved in WBA from an independent study had a sufficient intake of vitamin A, on average, at 1307.4 ± 725.6 μg/day, which meets the RDA. This value was much greater than that of nonathlete subjects, who averaged only 504 ± 29 μg/day, highlighting the added benefit of active participation in sports to nutrient intake (25).

#### 3.5.3 Vitamin C

Vitamin C plays a vital role in antioxidant protection, immune function, and tissue repair and may be particularly important for athletes subjected to oxidative stress from intense training (56). Among Brazilian para-athletes from different sports, the concentration ranges from 187.8–653.5 mg/day, which is much higher than the RDA of 90 mg/day for males and 75 mg/day for females, indicating strong antioxidant protection (56). Similarly, elite paramacyclists reported that the average vitamin C intake was 333 ± 430 mg/day for males and 383 ± 430 mg/day for females, which is related to high fruit and vegetable intake, which is very important in fighting oxidative stress during endurance training (Study 18, *N* = 31). Canadian Paralympic athletes also met vitamin C requirements, with males averaging 99 mg/day (123.7% of the RDA) and females consuming similar amounts, which highlights adequate dietary practices across nine sports (48). However, some deficiencies in vitamin C were noted. For example, in South African SCI endurance hand cyclists, while intake was adequate in general, with males having 140.4 mg/day (201.7% of the RDA) and females having 102.3 mg/day (136.4% of the RDA), dietary imbalances were noted because of unique metabolic challenges (61). In comparison, South Korean national Paralympic athletes demonstrated insufficient consumption levels at 70.9% of the RDA, indicative of restricted availability of fruits and vegetables (59). Additionally, a longitudinal investigation concerning wheelchair basketball athletes underscored that despite advancements ensuing from dietary counseling (DC), 89% of female participants and 78% of male participants continued to be below the recommended intake for vitamin C (82).

Interestingly, high consumption was also reported among Paralympic athletes living in Tabriz, Iran, where women consumed 477.72 ± 418.27 mg/day (637.18% of the RDA) and men 202.53 ± 200.62 mg/day (225.70% of the RDA), which is indicative of good dietary patterns (80). Meanwhile, Lithuanian deaf female basketball players were meeting their requirements with a mean daily intake of 174 mg, which approaches or fulfills recommendations (62). Nevertheless, a study revealed that, among Brazilian athletes, more than 35% failed to meet adequate levels of intake, which has been attributed to insufficient consumption of fruits and vegetables (*N* = 40) (32). Turkish wheelchair basketball players, although approaching adequate levels with a mean intake of 91.07 ± 71.83 mg/day, presented wide interindividual variability, suggesting that some are at risk of immune dysfunction (*N* = 36) (52). Overall, while most para-athletes exceeded the recommended amounts of vitamin C, there were some subgroups and instances of inadequacy. These findings underscore the importance of evaluating vitamin C intake in different para-athlete groups to ensure sufficient antioxidant protection and promote recovery.

#### 3.5.4 Vitamin E

As a potent antioxidant, vitamin E is known to be important in reducing the oxidative stress caused by exercise and has high variability in intake between Paralympic athletes of different sports and geographical regions (60). The Spanish National Wheelchair Basketball Team reported a suboptimal intake of vitamin E at 7.8 ± 2.5 mg/day, which was below the RDA of 15 mg/day due to low consumption of nuts and seeds (60). Similarly, the Canadian Paralympic athletes consumed only 59.4% of the RDA (7.13 mg/day), with both males and females showing suboptimal levels, potentially compromising antioxidant defenses during intense training (36).

In Lithuanian deaf female basketball players, the average vitamin E intake was 9.1 mg/day, which is close to adequate but with some differences from the recommended levels (83). Among Brazilian para-athletes, the mean intake of vitamin E was 11.2 mg/day (range: 7.9–18.2 mg/day), and individual athletes presented intakes below the RDA, thus having an uncertain potential to cope with oxidative damage under training loads (56). In contrast, elite South African SCI endurance hand cyclists revealed contrasting findings, with males and females exceeding the RDA through daily intakes of 15.1 mg/day and 18.1 mg/day, respectively, which may reflect robust antioxidant protection linked to structured dietary interventions (61). In contrast, Iranian Paralympic athletes reported intakes of 7.10 ± 5.98 mg/day (46.83% of the RDA for men) and 6.05 ± 4.76 mg/day (39.90% of the RDA for women), indicating considerable inadequacies across genders (80). A study of elite paracyclists competing at the international level revealed that females consumed 24 ± 16 mg/day, with 26% failing to meet the RDA, whereas males consumed an average of 46 ± 74 mg/day, often exceeding the upper limit due to supplementation (48). In wheelchair basketball athletes receiving dietary counseling, intake improved longitudinally, but 100% of females and 56% of males had inadequate vitamin E intake, emphasizing the need for long-term dietary interventions (82).

Among Iranian Paralympic athletes undergoing hemodialysis or those with hemophilia, vitamin E intake averaged 13.68 ± 7.09 mg/day, which is close to adequate and similar between the two groups, highlighting the importance of balanced diets rich in plant-based fats and antioxidants (81). In Korean national athletes with disabilities, vitamin E intake exceeded the RDA at 112% (14.57 mg/day) on average, indicating a positive dietary trend that fits the antioxidant protection needs (59). These studies present a wide spectrum of vitamin E intake among Paralympic athletes, which is influenced by factors such as dietary habits, access to nutrient-dense foods, supplementation practices, and the unique physiological demands of certain disabilities.

#### 3.5.5 Vitamin K

Vitamin K, which is crucial for blood clotting, plays a critical role in the performance and recovery of Paralympic athletes, particularly those at increased risk for bone fragility due to underlying medical conditions or intense physical activity (84). A study in a group of 100 Paralympic athletes undergoing hemodialysis reported adequate consumption of vitamin K, which is important for reducing the risk of osteodystrophy and bone metabolism (81). However, the intake of vitamin K differed among the populations studied. In a sample of 35 Paralympic athletes from Tabriz, Iran, the mean for male subjects was 92.07 ± 71.57 μg/day, which was equivalent to 76.25% of the Adequate Intake (AI), thus indicating potential inadequacy (80). Moreover, the mean intake of vitamin K among Korean national athletes with disabilities was slightly better, as it represented 88.8% of the Korean Dietary Reference Intakes (KDRIs); however, large interindividual differences indicate the need for individualized dietary interventions (59). These findings underscore the importance of personalized nutrition strategies in optimizing bone health and minimizing injury risk through the inclusion of foods rich in vitamin K, such as leafy greens. This is important for ensuring peak athletic performance and long-term skeletal integrity.

#### 3.5.6 Folate

Folate—an essential B-vitamin that participates in energy metabolism (32), DNA synthesis, and cellular repair—is, therefore, an indispensable nutrient for Paralympic athletes (32). The intake of vitamins generally remains inadequate across many populations, with particular disparities according to sex, type of sport practiced, and level of competition (52). A study conducted among 17 elite paracyclists reported that folate intake was markedly higher than expected as a consequence of widespread supplementation. The male participants in this study consumed an average of 726 ± 209 μg/day, whereas the female participants consumed an average of 727 ± 405 μg/day, both of which are significantly greater than the RDA (48). In contrast, a study using dietary records from 40 Canadian Paralympic athletes reported that male athletes consumed 92.9% of the RDA (370 μg/day) for folate, whereas female athletes consumed more than the RDA, with a consumption level of 117.2% (468.8 μg/day) (36). Similarly, a cohort study of 35 elite Paralympic athletes in Tabriz, Iran, reported a suboptimal intake of folate, with a mean value of 290.87 ± 139.62 μg/day, representing only 72.15% of the RDA (80). In another study on Brazilian para-athletes, specifically those enrolled in a sports scholarship program, significant folate inadequacies were observed, especially among nonscholarship athletes, who had less access to folate-rich foods (32). Although some athletes exceed the recommended folate levels through supplementation, others do not meet their needs. Thus, regular testing and individually tailored dietary interventions must be adopted to attain optimum folate intake, which underpins performance and health in such athletes.

#### 3.5.7 B vitamins

On the basis of several studies of the vitamin status of athletes with disabilities, B vitamins (thiamine, riboflavin, pyridoxine, folate, and cobalamin) are sufficient for energy metabolism, protein synthesis, immune function, and effectiveness health in athletes (1, 85). In general, when vitamin B1 (thiamine) intake was adequate; this in turn led to the production of sufficient energy, particularly for the duration of endurance sporting events (1) Research on Indonesian athletes (81) and Paralympic athletes with hemodialysis revealed that thiamine levels were within the normal range and were therefore able to support these athletes, who were in optimum metabolic function. Notably, riboflavin (B2) is present at satisfactory levels among an array of athletes, e.g., South African SCI endurance hand cyclists (71) and Paralympic athletes in Tabriz, Iran (80), where the vitamin aids proper energy production and helps with skin health. Vitamin B12, which supports the function of nerves and is involved in energy conversion, was generally supplied in sufficient amounts across all study groups, with South African SCI athletes among the others who did not show any signs of deficiency (61). As such, nerve function, in particular, is maintained in athletes with disabilities whose performance is dependent on remaining neurologically intact. Although the majority of them have shown an adequate level of B vitamins, some of them have vitamin deficiencies due to insufficient dietary diversity or specific sport-related needs. This finding highlights the importance of improving nutrition events specific to athletes to achieve optimal performance.

This review of the results of these studies highlights the role of B vitamins in both the physical and cognitive performance of disabled athletes by ensuring that the intake of both is optimal, although the effectiveness of some individuals remains an issue because they have special dietary needs.

### 3.6 Minerals

#### 3.6.1 Iron

Iron is a vital nutrient for Paralympic athletes since its role in oxygen transportation and energy metabolism is vital for optimal performance in endurance and strength-related sports (52). The prevalence of iron deficiency and inadequacy varies widely between athletes, and differences due to sex are one of the primary causes of concern.

Numerous studies have noted the major differences in iron intake among male and female athletes. For example, a study conducted on 66 Paralympic athletes from various sports, such as swimming, wheelchair basketball, and amputee football, indicated that female athletes had poor iron intake. On training days, they reportedly consumed an average of 9.6 mg/day, which was only 53% of the RDA. Non-training days had an even lower intake of 8.6 mg/day. In contrast, male athletes consumed an average of 9.8 mg/day on training days (122.5% of the RDA) and 9.4 mg/day on nontraining days, reflecting adequate iron intake (31). Another study revealed that iron deficiency occurs especially in female athletes, with 29.5% of them failing to meet iron intake recommendations, which increases the risk of developing anemia and negatively impacts their athletic performance (32). Ferritin levels, an indicator of iron status, are significantly different between sexes. A study of ferritin concentrations revealed a mean value of 56 ± 43 μg/L for females, whereas males averaged 145 ± 90 μg/L. Among the female athletes, three were classified as having iron deficiency without anemia, and two had iron deficiency anemia. In contrast, no iron deficiency or anemia was found among male athletes, because of changes in iron metabolism caused by exercise, inadequate dietary intake, or reduced energy consumption, which tends to aggravate the absorption of iron (71). One study demonstrated that, among wheelchair athletes, only 27.3% of female subjects met the RDA for iron. The average intake was 15.7 ± 4.2 mg/day. This especially underlines the need for special dietary interventions, especially in the case of female athletes, to avoid potential deficiencies that could detrimentally affect performance and increase levels of fatigue (25).

Studies exploring athletes with SCI have revealed that although the iron intake of male athletes meets certain requirements, 7–16% of female athletes are deficient. The intake of iron by female subjects ranged between 15.1 and 17.0 mg/day and provided an estimated average requirement (EAR) for nonpregnant females; however, the EAR still provided less than full levels for some athletes (63). These findings highlight the increased risk of iron deficiency anemia, particularly among female athletes, and suggest that individualized nutrition strategies and supplementation are important for maintaining peak performance and preventing fatigue.

#### 3.6.2 Calcium

Calcium is also important for both the health and performance of Paralympic athletes, since it not only maintains bone density but also has a role in muscle contraction and maintains the integrity of the skeleton (48). However, many studies have shown a persistent struggle to meet the RDA of 1,000 mg/day in this population, with significant variation influenced by sport, sex, and type of disability (60). One study carried out on Spanish wheelchair basketball players reported an average intake of calcium of 784 ± 211 mg/day, which is below the RDA. This deficiency is especially concerning athletes with SCI, as they are at increased risk of developing osteoporosis due to reduced mechanical loading (60). Similarly, a study of Canadian and American SCI athletes reported even lower intakes, with females consuming 541 ± 432 mg/day in autumn, which further declined to 462.3 ± 373.3 mg/day in winter. Male athletes showed slightly better levels, averaging 990.8 ± 589 mg/day in autumn and 869.7 ± 453 mg/day in winter, but still below the optimal thresholds for bone health (54). Across other populations, significant calcium deficiencies have been observed. For example, Indonesian swimming athletes in training for the ASEAN Para Games reported an average intake of 400 mg/day, which was only 36% of their RDA, reflecting very serious concerns about long-term skeletal health (27). Similarly, South African hand cyclists had critically low intakes—especially female athletes at 423.1 mg/day (42.3% of the RDA), compared with males at 890.2 mg/day (61). Turkish wheelchair basketball players averaged 749.72 ± 357.11 mg/day, just slightly above the lower end of the estimated average requirement (EAR) (28). Similarly, among Korean Paralympic athletes, intake reached only 55.74% of the RDA, emphasizing persistent gaps in dietary calcium (59).

Conversely, some athlete groups showed sufficient or even above-average calcium intake. For example, one study of international paramacyclists reported daily intakes of 1,558 ± 474 mg for males and 1,590 ± 608 mg for females, which exceeded the RDA and reflected a well-planned nutritional approach (48). In another study, Brazilian para-athletes also presented sufficient calcium levels, with males consuming 1637.5 mg/day and females consuming 1228.7 mg/day—levels necessary for bone integrity and muscular function (56). Lithuanian deaf female basketball players also met their RDA, averaging 1,026 mg/day, despite coexisting deficiencies in vitamin D (62). These disparities prove the critical requirement for individually targeted nutritional interventions, including dietary planning, fortified food options, and appropriate supplementation. Given the critical role of calcium in maintaining skeletal health, such a deficiency needs to be taken seriously to support performance and prevent long-term skeletal complications in these athletes, especially those with SCI or other disabilities.

#### 3.6.3 Zinc

Zinc itself is a major mineral involved in immune responses (48) and tissue regeneration (56) and is associated with overall health, especially in athletes. Many studies have examined zinc consumption/levels in various groups of Paralympic athletes, revealing large variations from one group to another regarding both zinc sufficiency and deficiency status (56). For example, a study of 39 elite athletes with SCI from Canada and the United States reported significant deficiencies, particularly due to inadequate energy intake. In autumn, female athletes ingested an average of 3.2 ± 2.7 mg/day of zinc, which showed a modest increase to 3.8 ± 4.8 mg/day during the winter months. In contrast, male athletes reported a zinc consumption of 9.0 ± 9.9 mg/day in autumn, which increased to 10.9 ± 11.4 mg/day in winter. Notably, these figures remained below the EAR for numerous participants, raising concerns regarding the athletes’ immune function and the processes of tissue repair (54). Similarly, a study among Indonesian swimming athletes with disabilities revealed an average intake of 3.6 mg/day of zinc that could only meet 36% of the RDA of 10 mg/day. This mineral is highly insufficient, especially in male and female athletes, since it plays an important role in wound healing and immune function (16). In contrast, a study of elite para-cyclists revealed much greater zinc intake, with male athletes consuming 25.6 ± 17.7 mg/day and female athletes consuming 29.6 ± 24.9 mg/day, both of which exceed the RDA values of 11 mg/day for men and 8 mg/day for women. This was likely due to their diet, which was rich in red meat and fortified cereals (48).

Additionally, 77.3% of the athletes in wheelchair basketball reported an adequate amount of zinc, with a mean of 11.5 ± 2.1 mg/day, indicating a more balanced dietary habit in this group. In any case, some athletes present with deficiencies; thus, particular attention should be given to specific groups within the population with respect to zinc intake (25). These findings clearly suggest a particular need to ensure adequate intake of zinc in special, high-risk groups, such as those with spinal cord injuries and female athletes.

#### 3.6.4 Magnesium

Magnesium is a crucial mineral in energy metabolism (54), muscle function (59) and bone health (86), and has been found to have unequal consumption levels between different groups of Paralympic athletes. Most of the available evidence suggests that there are significant inadequacies in magnesium intake because athletes in a variety of sports often fail to meet the RDA (59). One study on Spanish wheelchair basketball players indicated that magnesium intake was slightly below the RDA at 325 ± 71 mg/day, with a recommendation of 400 mg/day for male athletes. This may indicate that there is a gap in ensuring that magnesium levels are adequate, which might affect muscle relaxation and energy metabolism (60). Similarly, Canadian and American athletes who had suffered SCI had significantly lower magnesium intake, and on average, women consumed only 96.2 ± 70.9 mg/day and men 294.3 ± 293.6 mg/day during the fall season. These averages decrease even further in the winter months, which highlights concerns about inadequate energy availability and its impact on meeting micronutrient needs (54). Similarly, Indonesian swimming athletes with disabilities also showed suboptimal magnesium intake, averaging only 122 mg/day, which contributed to only 39% of the RDA of 310 mg/day for active individuals (27). In contrast, Brazilian para-athletes showed proper magnesium consumption of 615.8 mg/day on average, much higher than the RDA of 400–420 mg/day for males and 310–320 mg/day for females, indicating relatively better dietary intake (56). South African SCI hand cyclists also demonstrated sex differences in magnesium intake, with males consuming 115.6% of the RDA and females consuming only 88.2%, once more underlining the inadequate diets of this population (61). Most interestingly, a Korean study of athletes with disabilities revealed a significant deficiency in magnesium, accounting for 22.0% of the RDA, indicating that muscle function and recovery are compromised by this inadequacy (59).

#### 3.6.5 Iodine

Iodine is important for the maintenance of thyroid function and, by extension, metabolic regulation and energy balance, which are necessary for endurance performance (48). Iodine deficiency is an issue for many Paralympic athletes, since research shows severe inadequacies of dietary intake in a variety of sports. In a study of 17 elite paracyclists, for example, 33% of male athletes and 53% of female athletes reported iodine intakes below the RDA of 150 μg. On average, men consumed 130 ± 60 μg/day, whereas women consumed only 93 ± 71 μg/day. This study has shown the importance of including more iodine-rich foods, such as iodized salt and seafood, in the diets of these athletes to support their metabolic efficiency (48). Similarly, a study of 17 male wheelchair basketball players reported an average iodine intake of 110 ± 35 μg/day, which is close to the RDA but still below the optimal threshold. This finding indicates that some improvements in dietary practices may have resulted in a slight increase in iodine consumption (60). A study involving 31 elite paracyclists provides further support for the prevalent finding of iodine insufficiency. Male athletes averaged 130 ± 60 μg/day, whereas females had much lower intakes: 93 ± 71 μg/day, on average, accounting for only 87 and 62%, respectively, of the RDA. This is particularly important because of the role of iodine in thyroid hormone regulation and the other metabolic functions underlying athletic performance. This finding is consistent with worldwide concerns about the possibility of micronutrient deficiencies in athletes with disabilities, particularly those engaged in endurance sports, where metabolism regulation has become a major factor (48). However, not all studies report iodine deficiency. One study in 21 Korean national athletes with disabilities reported iodine intake of 143.2% of the RDA, suggesting that this cohort consumed sufficient iodine. This may be related to dietary practices that include adequate iodine-rich foods (59). These studies reveal a trend toward inadequate iodine status among many Paralympic athletes, particularly in the endurance sports category, where optimal thyroid function is essential for sporting performance (59).

#### 3.6.6 Potassium

Potassium plays a significant role in muscle function, fluid balance, and cardiovascular health (82). Potassium intake has been a nutritional concern among Paralympic athletes, as numerous studies have revealed critical inadequacy for their respective sports and gender groups. In particular, in a study conducted among wheelchair basketball athletes, the intake of potassium was low among those who did not have dietary advice; in fact, as many as 85% of males and 100% of females had below-threshold intakes (82). For Korean athletes who have disabilities, low potassium intake was identified by their intake of only 71.3% of the RDA (59). A Canadian Paralympic athlete cohort showed suboptimal consumption of potassium, with male athletes reaching only 66.2% and female athletes merely 57.3% of the established adequate intake levels (AIs). This could indicate a much broader trend in potassium deficiency, which is important for enhancing muscle function and potentially decreasing the risk of cramping or fatigue during high-intensity training sessions (36). One other study assessing Paralympic athletes in Iran reported similar patterns in which male athletes consumed only 70.95% of the AI for potassium and female athletes even lower, at 47.72%, highlighting that this deficiency is widespread across gender lines (80). In contrast, a study on Brazilian para-athletes revealed extremely good potassium intake levels, where athletes consumed an average of 5,594.3 mg/day, much higher than the RDA of 4,700 mg/day. This can be interpreted to mean that it is possible to greatly exceed the recommended intake of potassium with appropriate dietary strategies and guidance, which is beneficial for cardiovascular health, electrolyte balance, and overall muscular function (56). These findings highlight important nutritional strategies to limit potassium deficiency, particularly for the athlete population with disabilities, who have unique challenges in meeting nutritional needs to enhance performance and recovery in disabled athletes.

#### 3.6.7 Selenium

Selenium is a trace mineral known for its antioxidant properties and is important for immune function and metabolic health (1, 87); therefore, it is also an essential micronutrient for athletes with disabilities. Many studies evaluating the dietary intake of Paralympic athletes in different parts of the world provide meaningful information on trends in selenium intake and emphasize the importance of adequate intake to improve health and exercise performance (81). For instance, Canadian Paralympic athletes also presented very high levels of selenium, with males consuming 254% of the RDA (127 μg/day) and females consuming 190.5% (95.3 μg/day), which is much greater than what is recommended (36). A Brazilian study of high-performance para-athletes supported these observations, with an average intake of selenium of 235.2 μg/day, which is well above the RDA of 55 μg/day (56). Regarding selenium intake, a study examining 21 Korean national athletes with disabilities preparing for the Tokyo Paralympic Games reported that their selenium intake was, on average, 114.2% higher than the Korean recommended amount (59). These findings reflect a positive trend in the intake of selenium among Paralympic athletes, especially compared with other micronutrients such as magnesium and potassium, which generally result in higher rates of deficiency. Adequate consumption of selenium, as in the case of these studies, will help athletes keep the immune system functioning optimally, support recovery, and even enhance performance—all of which highlight the importance of tailored nutritional strategies, both individually and collectively.

#### 3.6.8 Phosphorus

Phosphorus is a critical mineral involved in energy metabolism, skeletal health, and cellular processes, making it especially important for athletes. Adequate phosphorus intake is necessary for maximal muscular performance, especially in elite athletic activities. However, similar to many other nutrients, a balanced intake of phosphorus is important, as both deficiencies and excesses can have negative consequences (1). Several studies investigating the knowledge and behavior of Paralympic athletes concerning their nutrition have shown optimal but also excessive phosphorus intake. For example, a study on a sample of 35 elite Iranian athletes reported that, according to the researchers who carried out that study, phosphorus intake was also extremely high in men (1262.20 ± 325.34 mg/day: 180% RDA) and female athletes (956.97 ± 258.84 mg/day: 136% RDA). Elevated levels of phosphorus consumption may positively influence bone health; however, excessive intake of phosphorus, especially when associated with insufficient calcium consumption, has the potential to interfere with calcium homeostasis, resulting in adverse consequences for bone mineralization and overall well-being (80). In a related study conducted in Turkey with 40 wheelchair basketball players, phosphorus intake was found to exceed the recommended levels for most athletes. The study revealed that phosphorus intake was remarkably high, especially in players whose diet mainly consisted of processed foods that usually contain high-phosphorus additives. This excessive consumption could result in the development of disorders in calcium metabolism, similar to those observed at inappropriate calcium-to-phosphorus ratios (52).

There may be concern regarding the phosphorus intake of some athletes, especially by those athletes on restrictive diets or those not meeting their daily nutrition requirements. Careful monitoring of phosphorus intake is thus highly advisable to avoid both deficiency and excessive intake, thereby providing athletes with the required intake for optimal development and performance.

#### 3.6.9 Sodium

Sodium intake among Paralympic athletes has always been a matter of significant concern, with a number of studies highlighting excessive and insufficient consumption across different populations. In a study of 21 Korean national athletes with disabilities, sodium intake reached more than double the AI level, reaching 216.6% of the RDA. The high consumption of sodium can negatively affect the intake of other essential nutrients and could result in longer-term implications, including damage to bone health, as sodium consumption impacts calcium retention (59). According to a study of 40 Canadian Paralympic athletes, nutrient intake varied significantly by sex; male athletes had a higher intake of several minerals on average. Sodium intake was consistently high, however, across both genders, which becomes a major contributing factor to cardiovascular risk, however, if it is carried out over a prolonged duration (36). In athletes from Iran, sodium intake has been established to be equivalent to 179.05% of the AI intake for males, with an average sodium consumption of 2662.85 ± 1367.07 mg/day, which is considered high and increasing concern about long-term health, especially cardiovascular health (80). High sodium intake was reported for wheelchair basketball athletes in Turkey, where sodium levels were reported to be 3827.82 ± 1,792 mg/day, exceeding the upper limits of the recommended intake range. Moreover, dependence on a diet of processed and salted foods again dominated as a significant contributor to high sodium intake (52). Despite the need for sodium during hydration, controlling its intake is crucial to avoid compromising long-term health (52). The data from these studies have shown the imperatives of nutrition personalization in Paralympic athletes by providing enough sodium to fulfill the need but not to exceed the recommended levels.

### 3.7 Micronutrient absorption and bioavailability in Paralympic athletes

A strong nutritional program focuses not only on diet but also on the level of micronutrient bioavailability and absorption that is attained by athletes during Paralympic competition. The Paralympic population often suffers from specific physiological dysfunctions that have a direct impact on the absorption of some vitamins and minerals, ensuring that even when dietary consumption has been achieved, deficiencies may be present (31).

#### 3.7.1 Gastrointestinal issues and absorption in athletes with SCI and CP

Spinal cord injury (SCI) and cerebral palsy (CP) increase the risk of developing gastrointestinal (GI) dysfunction in athletes, thus preventing the absorption of nutrients. SCI athletes with tetraplegia, in particular, are faced with problems such as gastroparesis or constipation, and as a result, their digestion is slowed, and these vitamins are poorly absorbed. Similarly, athletes with cerebral palsy, especially those with muscle tone abnormalities, can experience difficulties with swallowing and digestion, which in turn can disrupt the absorption of iron and calcium (14).

#### 3.7.2 The impact of physical activity on nutrient absorption

Intense physical activity increases the demand for minerals and vitamins due to increased metabolic rates. However, exercise-induced intestinal permeability (which is also known as “leaky gut” syndrome) can interfere with nutrient absorption, which is why it is critical for Paralympic athletes to strategically improve their nutrient intake and digestion. During intense training periods, magnesium, iron, and zinc are highly affected (14).

### 3.8 Micronutrient interactions in Paralympic athletes

In some cases, excessive intake of one micronutrient may interfere with the absorption or effectiveness of other micronutrients. For Paralympic athletes, some of the interactions among micronutrients should be addressed because they may affect their nutrient status and hence overall performance.

#### 3.8.1 Magnesium and calcium

Magnesium and calcium are also important for bone health and muscle function, but they may compete with each other in gastrointestinal absorption. Therefore, there needs to be a balance in their intake in athletes with SCI or amputations, where demand may be greater for both because of reduced mobility or weight-bearing activity. The ideal calcium-to-magnesium ratio is often 2:1, with athletes typically receiving enough magnesium (e.g., 200–400 mg/day) combined with their calcium needs (1).

#### 3.8.2 Iron and zinc

Both iron and zinc play a role in immune function and recovery; however, high levels of zinc can compromise iron absorption. This may be of special concern for athletes with SCI, as increased oxidative stress and inflammation may increase their need for these nutrients. High doses of zinc taken as supplements without adequate intake of iron can lead to exacerbation of deficiencies, especially in female athletes. Therefore, it is best that people take iron and zinc supplements at different times of the day to minimize the risk of absorption interference (1).

### 3.9 Micronutrient strategies for postexercise recovery

Postexercise recovery is an important aspect of training for Paralympic athletes. Adequate intake of micronutrients plays a critical role in muscle repair, immune function, and the reduction of inflammation—all of which are very important after intense training or competition.

#### 3.9.1 Vitamins C and E for recovery

Vitamins C and E are potent antioxidants that protect the body from oxidative damage related to physical activity. Both vitamins are thought to help reduce muscle inflammation and support tissue repair. Several studies have shown that taking vitamin C at doses of 200–500 mg daily can help reduce muscle damage and speed recovery in athletes participating in intense training, particularly athletes with SCI or amputations, who experience more pronounced oxidative stress during high-intensity activities (1, 36, 88).

#### 3.9.2 Magnesium and calcium for muscle function

Magnesium and calcium play crucial roles in how muscles work. Their deficiency can result in cramps and poor muscle recovery. In the case of athletes with amputations, the body may work differently, and the remaining limbs may experience more stress. Therefore, it is essential for them to consume enough magnesium and calcium. Magnesium supplements of 200–400 mg per day are taken for muscle recovery to reduce cramps. Similarly, calcium is needed for muscle contraction and stopping tiredness following exercise (1).

### 3.10 Support for immune health in Paralympic athletes

A strong immune system is important for athletes, especially those who train at high intensities. Intense exercise may temporarily suppress immune function, increasing the susceptibility of athletes to illness or injury (89, 90). In Paralympic athletes with SCI, amputations, and CP, the risk for infection or delayed recovery from injury is increased; special consideration should therefore be given to immune health through optimal intake of micronutrients (15).

#### 3.10.1 Immune function and vitamin D

Vitamin D plays an important role in immunological regulation, and insufficient amounts of vitamin D can damage the body’s ability to fight infection. Individuals with SCI or amputations are more likely to suffer from vitamin D deficiency due to limited sun exposure and impaired immune functions. Supplementing with 1,000–2,000 IU of vitamin D per day has been found to improve immune function and reduce the incidence of respiratory infections (1).

#### 3.10.2 Zinc to boost immunity

Zinc also plays a crucial role in maintaining the integrity of the immune system and supporting wound healing. Zinc deficiency is common in athletes with SCI, CP, and amputations, who may have compromised nutrition and increased levels of stress. Zinc supplementation (e.g., 10–15 mg/day) can help bolster immune function and decrease the duration of illnesses such as upper respiratory infections (1).

### 3.11 Liquid intake

Athletes’ performance and endurance in sports are significantly influenced by their level of hydration. Hydration status is influenced by several variables, including the type of activity, the athlete’s age, sex, muscle and fat mass and environmental conditions such as humidity and temperature (91). Liquid intake is important for Paralympic athletes, especially within recovery time, in addition to controlling bowel movement (52, 64). According to van de Vliet et al. (92), Paralympic athletes have similar water needs as healthy athletes do. Insufficient hydration status can cause heat-related illnesses that can endanger an athlete’s life in addition to their athletic performance and achievements (93, 94). Hypothermia or hyperthermia, for example, might manifest mild variations in body temperature in athletes who have had spinal cord injuries, affirming that athletes can avoid this circumstance by drinking enough fluids (95). Moreover, athletes are often advised to consume mineral water, electrolyte drinks, sports drinks, beverages with carbohydrates, and beverages with proteins (94). Drinks containing carbohydrates aid in providing energy fuel in the form of a solution that is simpler to absorb instantly to replace blood glucose and speeds up recovery (27). Milk and other protein-rich beverages are beneficial for repairing muscle throughout the healing process. The antioxidants included in fruit juices and vitamin C beverages help strengthen the body’s immune system (27).

A study conducted by Islamoglu et al. (52) reported that 40% of wheelchair basketball athletes ingested <0.5 L of liquids before the match, and 12.5% ingested >2 L of liquids daily. The liquid sources are predominantly water, mineral water, tea, coffee, fruit juice, and carbonated beverages. Additionally, a study conducted by Penggalih et al. (27) revealed that the average daily fluid consumption for swimming athletes with impairments was 3,222 ± 831 mL. Based on their classification system (27) where insufficient fluid intake is defined as less than 4,900 mL/day, the reported average intake was indeed insufficient. For context, general recommendations advise athletes to consume 150–300 mL of fluid every 15 to 20 min of exercise, with the volume varying depending on sweating rate (96). This approach would lead to a total intake of approximately 2,400–4,800 mL during a 4-h training session.

### 3.12 Supplementation

The goals of supplementation for Paralympic athletes include enhancing performance, providing energy, supporting recovery, and treating injuries (97). Supplements are used to address the nutritional challenges faced by physically and visually disabled para-athletes, including issues related to food preparation and practical difficulties such as preparing meals and feeding themselves (12, 98). Few studies have researched the impact of ergogenic aids and macronutrient supplements on physically disabled para-athletes, but in general, there is a dearth of evidence-based information regarding the risks, advantages, and proper usage of supplements for para-athletes (97, 99). Micronutrient supplements may be an option if optimal micronutrient intake cannot be met by regular nutritional intake. Before micronutrient supplements are recommended, potential reasons for deficiencies should be considered, such as conducting blood analyses and assessing dietary intake to confirm inadequacy. However, modifying the dietary intake of para-athletes is a preferred choice to avoid any adverse side effects of supplement use (27, 60).

Para-athlete supplement consumption has been estimated to be 85% higher than that of able-bodied athletes from different sports (100). A total of 64% of the 2004 Para Games Para athletes reported supplementation or medication consumption (101).

An observational study conducted with 65 Swiss wheelchair athletes from different sports reported that 63% of the athletes answered that they used supplements during training periods, and 43% used them before competitions (102). A further study conducted among 83 para-athletes who participated in the Tokyo 2020 Paralympic games and 30 para-athletes who participated in the Beijing 2022 Paralympic games revealed that the prevalence of supplement use in the year prior to competition was 56.7% among the Beijing 2022 Para athletes and 74.7% among the Tokyo 2020 Para-athletes (103). The two most popular supplements among para-athletes were protein powder (82.3 and 64.7%) and amino acids (85.5 and 76.5%) used by para-athletes at the Tokyo 2020 Paralympic Games and the Beijing 2022 Paralympic Games, respectively (103). Another study conducted among 12 South African Spinal Cord Injured endurance Hand Cyclists at National-Level supplement use reported rates of 40% before, 100% during, and 60% after training. Sports beverages were the most popular beverages during training, followed by energy gels and sports bars, but the protein powders Nutritional Practices and Body were the most popular beverages (61). In a cross-sectional study investigating supplement use in the previous 3 months among 42 wheelchair rugby athletes (33 males and 9 females) (WRAs), 90.9% of males and 77.8% of females reported using supplements, with vitamin D (26.2%), electrolytes (19.5%), and protein powder (19.5%) being the supplements most often taken. Performance was the most frequent justification for usage, followed by health (88). The common supplements used in 40 para-athletes included vitamin D, protein powder, sports bars, and drinks. Para athletes reported health, energy boost, medical reasons, performance enhancement, and recovery as the top five reasons for taking supplements, with percentages of 50.0, 42.5, 40.0, 37.5, and 37.5%, respectively. Males Para athletes primarily consumed protein and energy bars, protein powder, and sports beverages. On the other hand, females tended to consume vitamin D, protein powder, and then fatty acids in descending order. Males utilize more branched-chain amino acids (BCAAs) than females do (36). A study conducted among 399 adult para-athletes revealed that the main reasons for the use of nutritional supplements were immune support, exercise recovery, and energy. Multiple vitamins, sports drinks, protein, and carbohydrate supplements are the most widely used supplements. Athletes were asked if they had used supplements in the preceding 6 months, and 58% reported affirmative supplements. Elite athletes were more likely to utilize it, with 41% following the label directions for determining the appropriate dose and 9% having negative side effects following use (97). The most popular and reliable information source was a dietician, which is a positive outcome, followed by the coach and training partner; 52% of the athletes wanted more information/education about supplements, indicating that education on dosage and suitable sources of information is necessary and implying that supplemental information may be unobtainable or unreachable or that the athletes are unconcerned (97). Following the implementation of a pilot study including 14 male and female para-athletes with spinal cord injuries, the majority of the athletes followed the daily intake protocol of probiotic and prebiotic supplements for at least 80% of the days, and no significant adverse events occurred (104). It is possible to conduct a randomized controlled crossover trial (RCCT) to evaluate the impact of probiotic and prebiotic supplementation on spinal cord injury in Para athletes, as 71% of athletes said that they would be willing to participate in a similar study again. However, daily intake may still be challenging to achieve, particularly for athletes with full training and competition schedules. To solve this problem, study participation is scheduled during the noncompetition season. This will also decrease the possibility of unwanted adverse effects interfering with performance during competition (104).

Vitamin D status and supplementation were studied in case–control research among 84 elite Irish athletes, 33 of whom were para-athletes. The case group received vitamin D3 supplements during the winter (5,000 IU/day for 10–12 weeks and 50,000 IU once or twice, with doses scheduled at least 1 month apart) (105). Serum 25-hydroxyvitamin D tests were performed before and after supplementation for the case group and the control group. The results indicated that vitamin D deficiency was highly prevalent among elite Irish athletes and demonstrated that winter vitamin D3 supplementation efficiently preserves adequacy during winter and early spring while correcting any insufficiencies or deficiencies, with 25(OH)D significantly decreasing in those who were not given a vitamin D supplement (105).

Graham-Paulson et al. (106) performed a case study on caffeine supplements in an elite male para-triathlete that resulted in an increase in the performance of a 20 km time trial, where caffeine consumption at doses of 2, 4, and 6 mg/kg led to 2.7% faster time trial timings than did the placebo. This is likely due to increased arousal and higher power output at the same ratings of perceived exertion.

In a study on the effect of caffeine supplements, nine elite, physically disabled para-athletes participated in a placebo-controlled pilot study (98). The para-athletes completed a 1,500-meter time trial on a wheelchair training roller four times with four different treatments (placebo, caffeine, sodium citrate, and the combination of caffeine and sodium citrate) 1 h before the trial, and the time it took to complete the 1,500-meter exercise was not significantly different among the four treatments. Additionally, the performance of elite wheelchair athletes was not improved by supplementation with sodium citrate and/or caffeine (98).

## 4 Implications, recommendations, and future directions

Disabilities are considered one of the most important problems that occupy a vital position in human resource development programs (107). One of the most important requirements for their empowerment is providing all forms of social support, health care, and nutrition for people with special needs (73). There is no doubt that disability affects its owner, whether he was born with it or it appeared during his life as a result of an illness or accident, affecting his psychology, ability to achieve it, and his integration into society. The world of sports contributes to dismantling stereotypes by showing the diverse talents of people with disabilities. By communicating the exceptional performance of athletes with disabilities, thinking is often changed by encouraging an inclusive vision of society (4, 108).

Over the past few decades, Paralympics have become increasingly popular, with the most recent edition of Paralympics involving 4,400 athletes from over 100 countries competing for medals in 22 sports (8, 109). It also welcomes 10 different types of disability, far beyond those with spinal cord injuries (17). Paralympics now aim to empower athletes with disabilities to achieve sporting excellence and inspire and excite the world. Proper nutrition plays an important role in improving the lives of these Paralympic athletes. Nutritional approaches vary according to each of their needs, taking into account their health status, type of disability, age, and psychological state (110). The main goal of nutritional evaluation for para-athletes is to evaluate an individual’s eating habits to identify any deficiencies and provide any necessary adjustments to create the best possible diet plan (110, 111). Although there are many comprehensive studies on the effect of nutrition on athletes’ performance, studies on para-athletes are limited, and not much is known about nutritional needs. More scientific studies are needed to study the nutritional impact and nutritional needs of para-athletes. Adequate nutrition ensures energy recovery and the need to generate energy for this activity. Insufficient energy intake significantly shifts the energy balance to the negative side. An inadequate diet in terms of quality and quantity plays a role in the development of diseases that can lead to malnutrition and disability. For athletes with special demands, the primary goal of nutritional assessment is to ascertain intake, detect mistakes, and recommend adjustments to create the best possible nutritional plan (31, 110).

When developing a nutrition plan for athletes with special demands, factors such as energy usage, muscle fuel, hydration status, and assistance for energy generation should be considered. Depending on the area and type of physical disability, athletes have different dietary and energy demands. Although there are no standard values for daily recommended energy (DRI) and nutrient intake levels for athletes, athletes are similar to the general population and have a variety of nutrient requirements and compositions (48). Importantly, an appropriate nutritional program can be created by calculating an athlete’s specific requirements. The lower resting metabolic rate (RMR) and energy expenditure (REE) observed in para-athletes are attributed to lower muscle mass and potential autonomic nervous system alterations (46). Studies have shown that athletes with spinal cord disabilities (SCDs) have significantly lower RMRs than those with visual or hearing impairments (42). The RMR is an important component of total energy requirements, and ensuring energy balance is essential for maximizing the performance of athletes with disabilities. The limited number of studies conducted in this area has led to the inability to develop specific equations for athletes with disabilities. Therefore, accurate energy expenditure assessment for athletes with disabilities is crucial, often necessitating direct measurement methods such as indirect calorimetry, particularly given the potential for altered physiological responses like heart rate to affect the accuracy of predictive models (83).

Dietary programming for para-athletes is essential to ensure optimal bowel function and prevent gastrointestinal issues (24, 64, 110). Para-athletes experience high rates of constipation, making a high-fiber diet crucial for intestinal motility (52, 64). Frequent fiber ingestion is highly recommended for para-athletes with spinal cord injuries, as they may have specific gastrointestinal disorders. Para-athletes, including females and males, are advised to maintain a daily energy intake of approximately 32.6 kcal/kg of body mass/day, aligning with the recommended intake range of 1,500–2,300 kcal/day for elite athletes (51, 52). Furthermore, protein is necessary for hormonal production, tissue repair, and protein breakdown compensation, and para-athletes may consume less protein than those without disabilities because of their reduced muscle activity and energy needs (56). Research suggests that athletes should consume between 25 and 30% of their total energy content from fat (57, 58). Para-athletes with disabilities may have specific dietary requirements or face challenges in nutrient absorption due to their conditions; consequently, para-athletes have varying levels of vitamins and minerals. Insufficient hydration can lead to heat-related illnesses, such as hypothermia or hyperthermia, which can endanger an athlete’s life and athletic achievements (91–94). Para-athletes should consume mineral water, electrolyte drinks, sports drinks, carbohydrates, and proteins to maintain their hydration status.

Supplement use among para-athletes is prevalent, often exceeding that of their able-bodied counterparts, with common reasons including performance enhancement, energy, recovery, and addressing nutritional challenges related to their disabilities. While research on ergogenic aids in this population is limited, protein powder and amino acids are frequently used. Dietitians are identified as the most trusted source of information regarding supplementation, highlighting the need for further education in this area (97, 100, 101, 103).

Future research should focus on developing disability-specific nutritional guidelines based on larger, well-controlled studies. Longitudinal studies examining the long-term impact of nutritional interventions on the health and performance of para-athletes are also needed. Furthermore, research exploring the interplay between different types of disabilities, nutritional needs, and athletic performance would be valuable. Investigating the effectiveness of various nutritional strategies on recovery, injury prevention, and psychological well-being in this population warrants further attention.

## 5 Conclusion

This scholarly review has provided a comprehensive overview of the nutritional, energy, and fluid intake needs of para-athletes aged 18 and older. The findings highlight the critical need for individualized nutrition planning to optimize the performance and health of these athletes, considering the variability in energy expenditure, macronutrient and micronutrient requirements, and fluid balance. This review underscores the importance of continued research to establish evidence-based nutritional strategies that effectively address the diverse needs of para-athletes, including those with various disabilities.
