# Supplementary material for: Tailored nutrition strategies for Paralympic athletes: addressing unique energy, nutrients, and hydration needs to enhance performance and health
Source: Front Nutr. 2025 Jul 7;12:1572961. doi: 10.3389/fnut.2025.1572961 (PMC12277170; doi:10.3389/fnut.2025.1572961)
Supplement: Supplementary file 1 [file Table_1.docx]

Supplementary Material

# Supplementary Figure

**
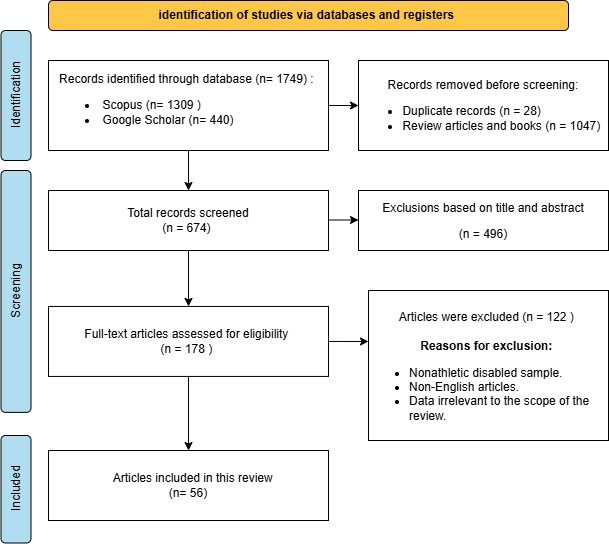
**

Figure 1. PRISMA diagram with data extraction process and eligibility assessment.

# Supplementary Table

**Table 1.** Comprehensive Synthesis of articles on Energy Availability, Nutritional Status, and Supplementation in Paralympic Athletes.

| **NO** | **Author** | **Year** | **Study Design** | **Energy, Macronutrient, Micronutrient, Supplement** | **Sample Size** | **Mean (Age ± SD)** | **Sex  Male%** | **Conclusion** |
| --- | --- | --- | --- | --- | --- | --- | --- | --- |
| 1 | Myoenzono K. | 2023 | cross-sectional | Dietary intake and supplement use | 1,392 | 36.1±9.7 | 75.8% | Paralympic athletes tend to have lower dietary quality and supplement use compared to Olympic athletes, with challenges like meal preparation and consumption and unique energy requirements impacting their diet. |
| 2 | Hertig-Godeschalk A. | 2023 | RCT | CHO, protein, fat | 14 | 34±9 | 43% | Two female athletes were found to have iron deficiency anemia, and the average vitamin D levels were inadequate. |
| 3 | Duarte Junior M.A. | 2023 | cross-sectional | CHO, protein, fat | 30 | 28.1±8.4 | 73.3% | Male para-athletes consumed more saturated fat than females, while younger athletes had higher protein intake (g/kg) than older ones. |
| 4 | Shaw K.A. | 2024 | cross-sectional | CHO, proteins, fats  sodium, cholesterol | 31 | 38 | 39% | Paracyclists generally meet or exceed nutrient recommendations, but some nutrients fall short, suggesting a need for more dietary variety. |
| 5 | Islamoglu A.H. | 2023 | cross-sectional | Energy, nutrient intakes, and EAR | 40 | N/A | 90% | Most basketball players consume inadequate amounts of CHO, fiber, thiamine, Ca, vit C, and vit D. Limited nutritional knowledge highlights the need for targeted nutrition education for wheelchair basketball players. |
| 6 | Weijer V.C.R. | 2024 | cross-sectional | RMR | 67 | 28±9 | 55.20% | Fat-free mass, total (T3) levels, and spinal cord disorders are key predictors of RMR in Paralympic athletes. This study's equations, along with those from Chun et al. and Nightingale & Gorgey, accurately predict RMR in this group. |
| 7 | Kahveci̇Oğlu T. | 2024 | cross-sectional | Energy, Macro, Micronutrient and hydration intake | 66 | 27.5±9.4 | 75.8% | Protein, CHO, iron, and Ca intakes fell below recommendations. CHO, vitamin D, Ca, and Mg levels were higher on training days, while hydration was inadequate regardless of gender, sport, or training status. |
| 8 | Weijer V.C.R. | 2024 | cross-sectional | Energy expenditure and nutritional intake | 48 | 27 | 39.50% | Para-athletes exhibit moderate to high energy expenditure, influenced by exercise duration and fat-free mass. |
| 9 | Toti E. | 2022 | cross-sectional | Energy, macronutrients,micronutrients, and fiber intakes. | 15 | 28.5±1.5 | 100% | Wheelchair basketball athletes (WBA) often regulate their eating to manage gastrointestinal symptoms and support well-being, including adherence to ON and the Mediterranean diet. |
| 10 | Deguchi M. | 2021 | cross-sectional | Dietary Practice and Nutrition Knowledge | 32 | 40.5±16.3 | 68.80% | Para-athletes had significantly lower nutrition knowledge than collegiate athletes. They displayed unique eating perceptions and limited knowledge, despite those practicing healthy eating reporting better body image. |
| 11 | Sukur A. | 2022 | cross-sectional | Energy intake, protein, carbohydrate, fat, and fluid intake. | 90 | N/A | N/A | Most athletes experience energy intake deficits, with CHO intake often insufficient, while fat and protein intake tend to be excessive. Fluid consumption ranges from 1000 to 8000 ml/day. |
| 12 | AL-Rubaye T.E.M. | 2022 | cross-sectional | Energy intake, macronutrient, micronutrient, fiber, cholesterol and Caffeine intake | 100 | 34.75±10.13 | 67% | Despite athletes in this study having anti-inflammatory DII scores, no significant link was found between DII and body composition parameters. |
| 13 | Madden R.F. | 2022 | cross-sectional | macronutrient, micronutrient and supplement intake | 80 | 35.9±8.4 | 61% | This study emphasizes the need for accurate sources and tailored nutrition education for para-athletes and coaches. |
| 14 | Gordon R.E. | 2022 | cross-sectional | Dietary and supplement intake | 12 | 44.0±9.3 | 83.33% | Intakes of vitamin D and calcium fell below RDA/AI for males. Supplement use was reported at 40% before, 100% during, and 60% after training. |
| 15 | Yokoyama H. | 2022 | A Pilot Study | Dietary challenges | 7 | 30–70 | 86% | Wheelchair para-athletes closely monitor calorie intake to maintain mobility, yet many lack routine dietary guidance from dietitians. Some see no need for such advice, often relying on personal notions of an “ideal diet” that may not enhance their performance. |
| 16 | Glisic M. | 2022 | RCCT | probiotic and prebiotic supplementation | 14 | 34±9 | 43% | Implementing a (RCCT) to assess the effect of prebiotic and probiotic supplementation is feasible in elite wheelchair athletes. |
| 17 | Jeoung B. | 2021 | cross-sectional | macronutrient and micronutrient intake | 21 | >18 | 76% | CHO and protein intake exceeded the %RDA, along with higher intakes of vit E, riboflavin, thiamine, B6, and B12. |
| 18 | Herrera-Amante C.A. | 2021 | Comparative Study | resting metabolic rate (RMR) | 15 | 18.7±6.5 | 53.33% | The Harris & Benedict equation showed the most consistency compared to indirect calorimetry. |
| 19 | Baranauskas M. | 2020 | cross-sectional | Energy Requirements, macronutrient and micronutrient intake | 14 | 26.4±4.5 | 0% | Deaf women athletes' diets are high in fat and saturated fats, increasing their risk of vitamin D and iron deficiencies. |
| 20 | Sasaki C.A.L. | 2021 | cross-sectional | Energy and micronutrient intake | 101 | 33.32±9.88 | 81.20% | The current Brazilian Federal sports scholarship program fails to adequately prevent micronutrient deficiencies in athletes. |
| 21 | Egger T. | 2020 | cross-sectional | Energy intake, Resting Energy Expenditure and Macronutrient Intake | 14 | 34.9±9.4 | 57.14% | Female athletes have a higher prevalence of low energy availability (LEA) than males, highlighting the need for increased energy intake to support energy demands and training adaptations. |
| 22 | Broad E.M. | 2020 | cross-sectional | resting energy expenditure (REE) | 14 | 31±6 | 100% | The measured REE of wheelchair rugby players was 1735 ± 257 kcal/day. Prediction models for individuals with and without SCI underestimated their REE, likely due to the athletes' higher REE relative to fat-free mass compared to less active groups. |
| 23 | Pelly F.E. | 2018 | case-control | resting energy expenditure (REE) | 7 | 31.3±7.3 | 100% | Existing prediction equations for estimating energy needs may need adjustment for athletes with spinal cord injuries. |
| 24 | Madden R.F. | 2018 | cross-sectional | Dietary Supplement | 42 | 36.3±9.5 | 78.60% | Supplement use among wheelchair rugby athletes was assessed, with performance and health being key motives. Future studies should examine nutrient intake and physiological levels to determine optimal supplementation techniques. |
| 25 | Joaquim D.P. | 2018 | cross-sectional | Energy Intake (EI), Energy Expenditure with Exercise (EEex) and Energy Availability (EA) | 17 | 26±6.17 | 53% | Paralympic athletes with high exercise energy expenditure (EEex) and insufficient or restricted energy intake (EI) should be closely monitored, as they risk developing low energy availability (LEA) and its effects. Currently, no specific LEA cut-off value exists for this group. |
| 26 | Joaquim D.P. | 2019 | cross-sectional | Dietary intake (food groups) | 20 | N/A | 65% | The diet quality of Brazilian Paralympic sprinters during a training camp requires improvements, particularly in wholegrain cereals, dairy, vegetables, and whole fruits. |
| 27 | Madden R.F. | 2017 | cross-sectional | Energy, macronutrient, micronutrient intake, and Supplement Use | 40 | 20.5–33.5 | 45% | Females fell short of the RDA for Fe and Ca, while males lacked vit A and B9. Common supplements included vit D, protein powder, sports bars, and drinks. |
| 28 | Eskici G. | 2016 | cross-sectional | Energy, macronutrient, micronutrient intake | 22 | 25.5±7.2 | 0% | This study revealed that some athletes consume insufficient energy, with low CHO intake (42.7%), high fat intake (44%), and inadequate levels of vit B1, B9, Mg, Fe, fiber, and fluids. |
| 29 | Ferro A. | 2017 | cross-sectional | Energy, macronutrient and water intake | 11 | 30±6 | 100% | Wheelchair basketball players expend less energy than able-bodied athletes but can enhance performance by increasing carbohydrate intake during main meals, especially around training sessions. |
| 30 | Grams L. | 2016 | longitudinal study | macronutrient and micronutrient intake | 17 | 30.0±6.5 | 100% | Elite Spanish wheelchair basketball players' overall energy intake is positively correlated with their micronutrient intake. A balanced diet with various food groups, especially nutrient-dense options is key to meeting micronutrient needs. |
| 31 | Flueck J.L. | 2017 | cross-sectional | supplement use, fluid and solid food intake | 65 | 39±12 | N/A | Swiss wheelchair athletes’ supplement uses closely mirrored that of general Paralympic athletes. The most common supplements included CHO, protein, multivitamins, minerals, recovery drinks, and ergogenic aids like creatine, caffeine, and beetroot juice. |
| 32 | Krempien J.L. | 2011 | cross-sectional | Energy, macronutrient and micronutrient intake | 32 | 30.6±6.2 | 75% | Vitamin and mineral supplements increased men's nutrient intakes but did not reduce inadequacy rates. These findings highlight those athletes with (SCI) are at risk of multiple nutrient inadequacies compared to the DRIs. |
| 33 | Goosey-Tolfrey V.L. | 2010 | cross-sectional | Energy, macronutrient and water intake | 23 | 25.1±8.1 27.6±7.2 | 39.10% | The energy from CHO, protein, and fat was similar for both F and M groups, though CHO intake was slightly below the recommended level for athletes. |
| 34 | Potvin, A. | 1996 | cross-sectional | Energy, macronutrient and micronutrient intake | 10 | 30.7 | 100% | Wheelchair athletes nearly met the recommended nutrient intake (RDI) for vitamins and minerals. |
| 35 | Aiello, Paola | 2023 | cross-sectional | Energy and macronutrients intake | 68 | 35.7±9.1 | 100% | Consistent with the link between body composition and energy balance, individuals with (SCI) and skill athletes have lower energy intake per kg of body mass and higher fat mass percentages compared to those in power, mixed metabolism, and endurance sports. |
| 36 | Gerrish, Heather R. | 2017 | cross-sectional | Energy, macronutrient and micronutrient intake | 39 | 21–47 | 51.30% | There was no significant difference in energy and macronutrient intake between groups based on the severity of the lesion. |
| 37 | Rastmanesh, Reza. | 2007 | case-control | Nutritional Knowledge | 72 | 30±7.6 | N/A | Iranian athletes with physical disabilities (APDs) lack essential nutritional knowledge to prevent health issues, particularly regarding disability-specific needs. |
| 38 | Penggalih, Mirza. | 2019 | Descriptive | Energy, macronutrients, micronutrients, and fluid intake | 18 | 25 | 88.90% | The disabled swimmers' body fat percentage and somatotype differ from professional swimmers' standards, while their energy, nutrient, and fluid intakes fall short of dietary recommendations. |
| 39 | Schneider, Willian VD | 2023 | cross-sectional | Diet quality | 101 | N/A | 81.20% | Both BHEI-R and GDQS effectively assess diet quality in athletes with disabilities. |
| 40 | Pritchett, Kelly | 2021 | cross-sectional | Energy availability and macronutrient intake | 18 | 27±7 | 50% | Calculated energy availability (EA) suggests a low risk of RED-S, but hormonal data indicate a high risk in this para-athlete group. |
| 41 | Juzwiak, Claudia Ridel | 2016 | Comparative Study | Basal Metabolic Rate | 30 | 26.5 | 63.33% | The Owen and Mifflin equations most accurately predicted BMR for these athletes. |
| 42 | Pegorin, Gabriela Rocha | 2020 | case study | Energy expenditure with exercise | 5 | 23.6 | 40% | Athletes with visual impairments showed EEEx levels from light to vigorous intensity. |
| 43 | Sanz-Quinto, Santiago | 2019 | case report | nutritional intervention | 1 | 36 | 100% | The elite wheelchair marathoner training at 3860 m needs higher nutrient intake and careful adjustments to their nutrition plan. |
| 44 | Graham-Paulson, Terri Susan | 2015 | cross-sectional | nutritional supplements | 399 | ≥18 | 74% | The main reasons for using nutritional supplements were exercise recovery, immune support, and energy. |
| 45 | Portela, Catarina Aires | 2024 | cross-sectional | nutrition knowledge | 36 | 29.8±12.7 | 72.20% | Athletes’ and coaches’ nutrition knowledge must be improved. |
| 46 | Gordon, Reno Eron | 2023 | cross-sectional | nutrition-related challenges | 12 | N/A | 83.33% | Bowel and bladder problems, restricted hand function, muscle spasms, thermoregulation problems, pressure sores, menstruation, and low iron/anemia were among the physiological and nutritional difficulties that hand cyclists reported. These factors were observed to decrease exercise capacity and restrict food and fluid intake. |
| 47 | Toti, Elisabetta | 2021 | cross-sectional | Energy, macronutrients, and micronutrient intake | 61 | 25.4 | N/A | Wheelchair basketball athletes improved their diet following dietary advice but did not follow the EAT-Lancet recommendation of high red meat and low legumes. |
| 48 | Magee, Pamela | 2013 | RCT | Vitamin D supplementation | 84 | ≥18 | 89.30% | This study found a high prevalence of vit D deficiency among Irish elite athletes, which also demonstrated that vit D3 supplementation in the winter and early spring effectively maintains sufficiency. |
| 49 | Rodrigues, Dayane Ferreira | 2018 | longitudinal study | Energy and macronutrients intake | 10 | 29.1±6.06 | 60% | During the three periods, there were notable differences in body mass, food intake, skinfold thickness, and macronutrients. The most common time for nutritional deficits to emerge is during the pre-competition phase. |
| 50 | Amirsasan, Ramin | 2017 | cross-sectional | Vitamin and Mineral Intakes | 35 | 37.17±8.69 35.64±12.38 | 68.60% | Many Paralympic athletes have lower-than-recommended intake of key vitamins and minerals, which are crucial for muscle repair, recovery, and performance. Athletes should focus on improving their diet and food choices. |
| 51 | Krempien, Jennifer Luella | 2012 | cross-sectional | Energy and macronutrient intake | 32 | 30.6±6.2 | 75% | Athletes with spinal cord injuries face a unique energy balance challenge, balancing energy intake, needs, and expenditure during physical training. They may monitor or restrict their diet to prevent obesity. |
| 52 | Graham-Paulson, Terri | 2018 | case study | caffeine | 1 | 46 | 100% | Caffeine enhanced the 20 km TT performance of an elite male Para-triathlete, likely due to increased arousal and higher power output at the same RPE. |
| 53 | Gawlik, Krystyna | 2015 | cross-sectional | lipid profile | 32 | 29±9.03 | 100% | The study participants had a lower percentage of overweight and obese men compared to the general male population in Poland, along with a better lipid profile. |
| 54 | Kelly Pritchett | 2016 | longitudinal study | Vitamin D | 39 | 27.7±6.5 | 48.70% | In the autumn and winter, a significant percentage of top athletes with SCI had deficient (15.4%) and insufficient (41%–51%) 25(OH)D levels. However, this investigation did not find any seasonal drop in vitamin D level. |
| 55 | Flueck JL | 2014 | RCT | caffeine and sodium citrate | 9 | 28 | 66.67% | There was no ergogenic effect of caffeine and/or sodium citrate supplementation on the 1,500-meter workout performance of wheelchair top athletes. |
| 56 | Spendiff, Owen | 2005 | RCT | Glucose drink | 8 | 31+5 | 100% | Both high and low carbohydrate intake prior to exercise did not result in a hypoglycemic reaction or negatively impacted exercise and performance in a group of paraplegic athletes with modest lesion severity. |
